# Comparative efficacy and safety of traditional Chinese medicine injections combined with chemotherapy for advanced non-small cell lung cancer: a Bayesian network meta-analysis

**DOI:** 10.3389/fimmu.2026.1901595

**Published:** 2026-08-19

**Authors:** Zhan-ze Ma, Yu-meng Wei, Shu-chun Yu, Mo-han Liu, Yue-feng Cheng, Bo-yi Wang, Rui-ge Sun

**Affiliations:** 1 Department of Integrated Traditional Chinese and Western Medicine, Liaoning University of Traditional Chinese Medicine Xinglin College, Shenyang, China; 2 Department of Traditional Chinese Medicine, Liaoning University of Traditional Chinese Medicine Xinglin College, Shenyang, China; 3 Oncology Department, The Fourth People’s Hospital of Shenyang, Shenyang, China; 4 State Key Laboratory of Organ Regeneration and Manufacturing, Institute of Zoology, Chinese Academy of Sciences, Beijing, China; 5 Third Internal Medicine Department, Dalian Hospital of Traditional Chinese Medicine, Dalian, China; 6 Organization Department, Liaoning University of Traditional Chinese Medicine, Shenyang, China; 7 Integrated Traditional Chinese and Western Medicine Oncology Department, Shenyang Fifth People Hospital, Shenyang, China

**Keywords:** Bayesian network meta-analysis, chemotherapy, immunomodulation, natural killer cells, non-small cell lung cancer, T lymphocytes, traditional Chinese medicine injections, tumor markers

## Abstract

**Background:**

Non-small cell lung cancer (NSCLC) remains a leading cause of cancer-related mortality worldwide. Although chemotherapy is an important treatment strategy for advanced NSCLC, its efficacy is limited by treatment resistance and adverse events. Traditional Chinese medicine injections (TCMIs) are widely used as adjunctive therapies; however, the comparative efficacy and safety of different TCMI-based regimens remain unclear. This study aimed to compare the efficacy and safety of various TCMIs combined with chemotherapy using Bayesian network meta-analysis.

**Methods:**

RCTs on TCMIs combined with chemotherapy for NSCLC were retrieved from Chinese databases including CNKI, Wanfang, VIP, and Chinese Biomedical Literature Database, as well as English databases including PubMed, Web of Science, and Cochrane Library. The retrieval period was from January 1, 2021 to May 1, 2026. The Cochrane risk-of-bias tool was used to assess the methodological quality of the included studies, and the certainty of network evidence was evaluated using the Confidence in Network Meta-Analysis (CINeMA) framework. Stata and R software were used to perform network meta-analysis.

**Results:**

A total of 71 RCTs involving 6,386 patients were included. The results of the network meta-analysis showed that Kanglaite Injection combined with chemotherapy showed a relatively favorable ranking for objective response rate (ORR), CD8, and skin adverse reactions; Kang’ai Injection combined with chemotherapy for hemoglobin reduction-related adverse reactions; Shenqi Fuzheng Injection for natural killer cells (NK cells), immunoglobulin G (IgG), neuron-specific enolase (NSE), and carbohydrate antigen 19-9 (CA19-9); Aidi Injection for immunoglobulin A (IgA), immunoglobulin M (IgM), and bone marrow suppression-related adverse reactions; Fufang Kushen Injection for carcinoembryonic antigen (CEA), vascular endothelial growth factor (VEGF), and adverse reactions such as leukopenia, liver and kidney function damage, and gastrointestinal symptoms; Shenmai Injection combined with chemotherapy for carbohydrate antigen 125 (CA125); Yadanzi Youru injection combined with chemotherapy for squamous cell carcinoma antigen (SCC); Xiao’aiping Injection for cluster of differentiation 4 positive T lymphocyte (CD4^+^ T Cell)/cluster of differentiation 8 positive T lymphocyte (CD8^+^ T Cell); Renshen duotang injection for disease control rate (DCR), cluster of differentiation 3 positive T lymphocyte (CD3^+^ T Cell), cluster of differentiation 4 positive T lymphocyte (CD4^+^ T Cell), and cytokeratin 19 fragment (CYFRA21-1); Huangqi Duotang injection combined with chemotherapy for thrombocytopenia-related adverse reactions. Meta-regression analyses identified statistical associations between age and effect estimates of CYFRA21-1, SCC, VEGF, and IgM; however, these findings should be interpreted as exploratory associations rather than causal explanations. Subgroup analysis of CYFRA21–1 suggested potential differences across age-related subgroups, which require further validation.

**Conclusion:**

TCMIs combined with chemotherapy maybe associated with improvements in clinical response, immune-related indicators, tumor-associated markers, and treatment tolerability in patients with advanced NSCLC. However,given the limited certainty of evidence, substantial heterogeneity, and methodological limitations of included studies, these findings should be interpreted cautiously and require confirmation through high-quality prospectivestudies.

**Systematic Review Registration:**

https://www.crd.york.ac.uk/PROSPERO/, identifier CRD420261392216.

## 1 Introduction

Globally, lung cancer remains the leading cause of cancer incidence and mortality among all malignant tumors (1). Non-small cell lung cancer (NSCLC) is the predominant histological subtype of lung cancer, accounting for approximately 80%–90% of all lung cancer cases, and is also one of the major causes of cancer-related death worldwide (2). In recent years, with the acceleration of population aging and continuous exposure to risk factors such as smoking and air pollution, the incidence and mortality of NSCLC have continued to rise (3, 4). Particularly in China, the disease burden of NSCLC has become increasingly severe and has emerged as a major public health problem threatening human health. Due to its insidious onset and lack of specific clinical manifestations in the early stage, most patients are diagnosed at stage III–IV and consequently lose the opportunity for radical surgical resection. Therefore, chemotherapy-based comprehensive treatment remains one of the core therapeutic strategies for advanced NSCLC (5).

At present, platinum-based doublet chemotherapy regimens remain the fundamental treatment strategy for advanced NSCLC, including commonly used regimens such as gemcitabine plus cisplatin (GP), paclitaxel plus cisplatin (TP), docetaxel plus cisplatin (DP), and vinorelbine plus cisplatin (NP). Numerous studies have demonstrated that chemotherapy can inhibit tumor cell proliferation to a certain extent, delay disease progression, and improve clinical symptoms. However, the clinical benefits of chemotherapy alone remain limited. On the one hand, the objective response rate (ORR) and disease control rate (DCR) are still unsatisfactory, and some patients are prone to developing drug resistance and disease progression during treatment. On the other hand, chemotherapy-related toxic and side effects are prominent, including myelosuppression, gastrointestinal reactions, immune dysfunction, and liver and kidney injury, which not only impair patients’ quality of life but may also reduce subsequent treatment tolerance and compliance. Therefore, improving antitumor efficacy while alleviating chemotherapy-related toxicity and enhancing immune function has become an important research direction in the comprehensive treatment of advanced NSCLC.

In recent years, the combination of traditional Chinese medicine injections (TCMIs) with chemotherapy has gradually attracted widespread attention in the treatment of advanced NSCLC. Modern pharmacological studies have shown that various TCMIs possess multiple pharmacological effects, including antitumor activity, immunoregulation, anti-inflammatory effects, and improvement of the tumor microenvironment. Relevant studies have demonstrated that TCMIs can inhibit tumor cell proliferation and invasion, induce tumor cell apoptosis, and suppress tumor angiogenesis by regulating signaling pathways such as phosphatidylinositol 3-kinase/protein kinase B (PI3K/Akt), nuclear factor-kappa B (NF-κB), and Janus kinase/signal transducer and activator of transcription (JAK/STAT) (6). Meanwhile, TCMIs can also improve immune function by increasing the levels of cluster of differentiation 3 positive T lymphocyte (CD3^+^; T Cell), cluster of differentiation 4 positive T lymphocyte (CD4^+^; T Cell), and natural killer (NK) cells, as well as regulating the CD4^+^; T Cell/CD8^+^; T Cell ratio, thereby enhancing antitumor immune responses. In addition, TCMIs have shown certain advantages in reducing chemotherapy-related adverse reactions, including nausea and vomiting, myelosuppression, and immunosuppression, thereby improving overall treatment tolerance and quality of life in patients.

Further studies have revealed that the occurrence and progression of advanced NSCLC is a complex pathological process involving multiple mechanisms, including abnormal tumor cell proliferation, persistent activation of chronic inflammatory responses, immune escape, imbalance of the tumor microenvironment, and abnormal expression of tumor-related factors (7). Tumor markers such as carcinoembryonic antigen (CEA), cytokeratin 19 fragment antigen 21-1 (CYFRA21-1), neuron-specific enolase (NSE), and vascular endothelial growth factor (VEGF) can reflect tumor burden and disease progression to a certain extent (8, 9). Meanwhile, immune indicators including CD3^+^; T Cell, CD4^+^; T Cell, CD8^+^; T Cell, and NK cells can reflect the immune status of patients (10). Therefore, comprehensively evaluating different therapeutic regimens from multiple dimensions, including tumor response, immune regulation, improvement of tumor markers, and treatment safety, is of great significance for comprehensively assessing the clinical value of TCMIs combined with chemotherapy.

Currently, a large number of randomized controlled trials (RCTs) have reported the potential advantages of TCMIs combined with chemotherapy in the treatment of advanced NSCLC. However, several limitations still exist in the current evidence, including relatively small sample sizes, inconsistent study quality, differences in the types of TCMIs and chemotherapy regimens used across studies, and the lack of direct comparisons among different TCMIs. In addition, traditional meta-analysis can only provide direct comparisons between two interventions and is unable to simultaneously evaluate the relative efficacy differences and ranking of multiple treatment regimens.

With the rapid development of immune checkpoint inhibitors (ICIs), the therapeutic landscape of advanced NSCLC has undergone substantial changes, and immunotherapy-based combinations have become an important component of current clinical practice. However, the evidence synthesized in the present study primarily derives from chemotherapy-based treatment settings, and therefore should be interpreted within this specific therapeutic context. Although the potential role of TCMIs in combination with ICIs remains to be further investigated, the current evidence may provide complementary information regarding the optimization of supportive and adjunctive strategies for patients receiving systemic therapy.

Based on the above background, the present study systematically searched relevant domestic and international literature and included randomized controlled trials investigating TCMIs combined with chemotherapy for advanced NSCLC. A Bayesian network meta-analysis (NMA) was conducted to comprehensively compare the efficacy and safety of different TCMIs combined with chemotherapy. The analysis focused on multiple outcomes, including ORR, DCR, immune function indicators, tumor markers, and adverse reactions. Furthermore, the surface under the cumulative ranking curve (SUCRA) was used to rank different interventions, with the aim of providing more reliable evidence-based evidence for the optimization of clinical treatment strategies and rational drug use in advanced NSCLC.

## 2 Materials and methods

### 2.1 Study design and registration

This study was conducted as a systematic review and Bayesian NMA to comprehensively compare the efficacy and safety of different TCMIs combined with chemotherapy in the treatment of advanced NSCLC. The study protocol was prospectively registered on the International Prospective Register of Systematic Reviews (PROSPERO) prior to formal literature screening and data extraction, with the registration number CRD420261392216. The entire study process was designed and reported in accordance with the Preferred Reporting Items for Systematic Reviews and Meta-Analyses (PRISMA) statement and its extension for NMA. All analyses were conducted using R software (version 4.3.1) with the ‘netmeta’ and ‘gemtc’ packages, ensuring reproducibility and transparency. Risk of bias assessment for included studies followed the Cochrane RoB 1.0 tool, while certainty of evidence was evaluated using the Confidence in Network Meta-Analysis (CINeMA) framework. As a systematic review and NMA, the present study synthesized data from previously published RCTs and did not directly recruit participants or administer clinical interventions.

### 2.2 Literature search strategy

A systematic literature search was performed in the China National Knowledge Infrastructure (CNKI), Wanfang Database, VIP Database, Chinese Biomedical Literature Database (CBM), PubMed, Web of Science Core Collection, and Cochrane Library for randomized controlled trials investigating TCM injections combined with chemotherapy for advanced NSCLC. The search period ranged from January 1, 2021 to May 1, 2026.

A combination of subject headings and free-text terms was used for literature retrieval. The search strategy mainly included: (1) disease-related terms, such as “non-small cell lung cancer”, “advanced NSCLC”, “metastatic NSCLC”, “lung adenocarcinoma”, and “lung squamous cell carcinoma”; (2) intervention-related terms, including “traditional Chinese medicine injection”, “Aidi Injection”, “Fufang Kushen Injection”, “Kanglaite Injection”, “Shenqi Fuzheng Injection”, and “Huachansu Injection”; (3) chemotherapy-related terms, such as “chemotherapy”, “platinum-based chemotherapy”, “combined chemotherapy”, “GP regimen”, “TP regimen”, “NP regimen”, and “DP regimen”; and (4) study-type terms, including “randomized controlled trial”, “randomized”, and “clinical trial”.

**Figure 1:**
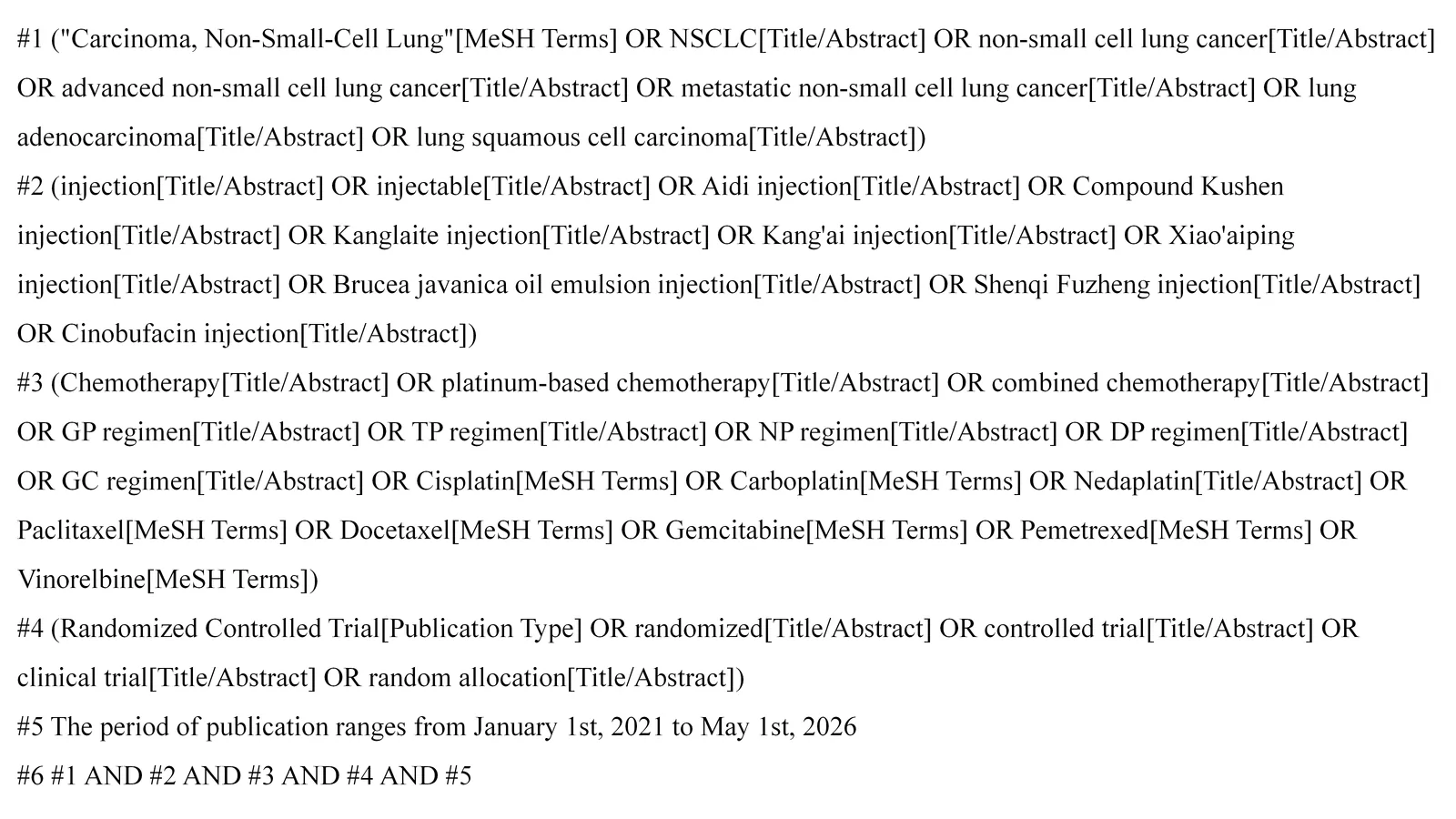
PubMed literature search strategy.

The search strategy was appropriately adjusted according to the characteristics of different databases. In addition, the reference lists of relevant reviews and included studies were manually screened, and gray literature, conference proceedings, and dissertations were also searched to minimize publication bias and reduce the possibility of missing eligible studies. Taking pubMed as an example, after literature retrieval, the results were imported into NoteExpress for literature management (**Figure 1**).

### 2.3 Eligibility criteria

The inclusion and exclusion criteria were formulated according to the PICOS principle.

#### 2.3.1 Inclusion criteria

Studies meeting all of the following criteria were included: (1) Participants: Patients pathologically or cytologically diagnosed with stage III–IV NSCLC, including lung adenocarcinoma, lung squamous cell carcinoma, and advanced/metastatic NSCLC, regardless of sex or ethnicity; (2) Interventions: The experimental group received TCM injections combined with chemotherapy. TCM injections included Aidi Injection, Compound Kushen Injection, Kanglaite Injection, Shenqi Fuzheng Injection, Huachansu Injection, and other commonly used TCM injections. Chemotherapy regimens were not restricted and included platinum-based chemotherapy, combination chemotherapy, and conventional chemotherapy regimens; (3) Comparators: The control group received the same chemotherapy regimen alone as that used in the experimental group; (4) Outcomes: At least one of the following outcomes was reported: ORR or complete response (CR) plus partial response (PR), DCR or CR plus PR plus stable disease (SD), immune function indicators (CD3^+^; T Cell, CD4^+^; T Cell, CD8^+^; T Cell, CD4^+^; T Cell/CD8^+^; T Cell ratio, NK cells), immunoglobulin indicators (IgA, IgG, IgM), tumor marker indicators (CEA, CYFRA21-1, NSE, cancer antigen 125 [CA125], cancer antigen 19–9 [CA19-9], squamous cell carcinoma antigen [SCC], and vascular endothelial growth factor [VEGF]), or incidence of adverse reactions; (5) Study design: RCTs published in Chinese or English.

#### 2.3.2 Exclusion criteria

Studies meeting any of the following criteria were excluded: (1) Non-randomized studies, retrospective studies, case reports, reviews, meta-analyses, or animal experiments; (2) Studies involving patients with small cell lung cancer, stage I-II NSCLC, or other pulmonary malignancies; (3) Studies in which additional traditional Chinese medicine therapies, targeted therapies, or immunotherapies were simultaneously administered in the experimental group, making it impossible to independently evaluate the efficacy of TCM injections; (4) Duplicate publications, studies with incomplete data, or studies from which relevant outcome data could not be extracted; (5) Studies with obvious methodological defects or inconsistent reporting of results.

### 2.4 Literature screening and data extraction

Two investigators independently screened the retrieved studies following a three-step process consisting of title screening, abstract screening, and full-text evaluation. Any disagreements were resolved through discussion or consultation with a third investigator.

A standardized data extraction form was designed before formal extraction. The extracted information included first author, publication year, sample size, patient age, pathological subtype, TNM stage, intervention details, chemotherapy regimens, treatment duration, outcome indicators, and adverse events. In addition, information related to methodological quality assessment, including randomization methods, allocation concealment, blinding implementation, and completeness of outcome data, was also collected. After data extraction, the two investigators cross-checked all extracted information to ensure data accuracy and consistency.

### 2.5 Outcome measures

To comprehensively evaluate the clinical value of TCM injections combined with chemotherapy in advanced NSCLC, outcome indicators were classified according to their clinical significance.

Primary outcomes included ORR, DCR, and incidence of adverse reactions. ORR and DCR were evaluated according to the response evaluation criteria in solid tumors. Secondary outcomes included immune function indicators (CD3^+^ T Cell, CD4^+^ T Cell, CD8^+^ T Cell, CD4^+^ T Cell^/^CD8^+^ T Cell ratio, and NK cells), humoral immune indicators (IgA, IgG, and IgM), and tumor marker indicators (CEA, CYFRA21-1, NSE, CA125, CA19-9, SCC, and VEGF).

A multidimensional evaluation framework integrating tumor response, immune regulation, tumor burden, and treatment safety was established to comprehensively assess the therapeutic advantages of different TCMIs regimens combined with chemotherapy.

### 2.6 Risk of bias assessment

The methodological quality of the included studies was independently evaluated by two investigators using the Cochrane Risk of Bias assessment tool. The assessment domains included random sequence generation, allocation concealment, blinding of participants and personnel, blinding of outcome assessment, completeness of outcome data, selective reporting, and other potential sources of bias. Each domain was judged as “low risk”, “high risk”, or “unclear risk”. Any disagreements were resolved through discussion or adjudication by a third investigator. The final risk of bias results were visualized using Review Manager 5.4 software.

In addition, the certainty of evidence for each network comparison was assessed using the CINeMA framework. The assessment considered six domains, including within-study bias, reporting bias, indirectness, imprecision, heterogeneity, and incoherence. Based on the level of major or minor concerns across these domains, the certainty of evidence was classified as high, moderate, low, or very low. Given that the evidence networks for some outcomes lacked closed loops, direct assessment of inconsistency between direct and indirect evidence was not feasible; therefore, the incoherence domain was evaluated cautiously. Furthermore, because most included studies lacked information on allocation concealment, blinding, and prospective trial registration, concerns regarding within-study bias and reporting bias resulted in downgrading the certainty of evidence for the affected comparisons in accordance with the CINeMA framework. The CINeMA assessment was performed independently by two reviewers, and any disagreements were resolved through discussion or consultation with a third reviewer.

### 2.7 Statistical analysis

R software, STATA 17.0, and OpenBUGS software were used for statistical analysis. Odds ratios (ORs) with 95% confidence intervals (CIs) were used as effect sizes for dichotomous variables, while mean differences (MDs) with 95% CIs were used for continuous variables. A Bayesian random-effects network meta-analysis based on Markov chain Monte Carlo (MCMC) methods was performed to improve adaptability to heterogeneity among studies. Three Markov chains with different initial values were simultaneously run, and convergence of the model was assessed using the potential scale reduction factor (PSRF). A total of 3 Markov chains were set, with 1000 adaptive iterations (n.adapt), 1000 burn-in iterations (n.burnin), and a total of 10000 iterations (n.iter). After excluding the burn-in period samples, the effective posterior sample size was 9000, with a thinning interval of 1. Evidence network plots were constructed, and consistency was assessed using both global and local methods.

Network evidence plots were generated to illustrate the direct and indirect comparisons among interventions. The node-splitting method was used to evaluate the consistency between direct and indirect evidence. When P > 0.05, consistency was considered acceptable and the consistency model was adopted; otherwise, potential sources of heterogeneity and inconsistency were further explored.

The SUCRA was calculated to rank the efficacy and safety of different TCMIs combined with chemotherapy. A higher SUCRA value indicated a greater probability that the intervention was among the optimal treatment regimens.

Heterogeneity among studies was assessed using the I² statistic. When substantial heterogeneity was identified, sensitivity analysis or subgroup analysis was further performed to explore potential sources of heterogeneity. When at least 10 studies were included for a specific outcome, comparison-adjusted funnel plots and Egger’s regression analysis were used to evaluate publication bias.

No additional adjustment for multiple comparisons, such as false discovery rate (FDR) correction, was applied because treatment effects were estimated within the Bayesian network meta-analysis framework by integrating direct and indirect evidence rather than through multiple independent hypothesis tests.

## 3 Results

### 3.1 Literature search results

**Figure 2:**
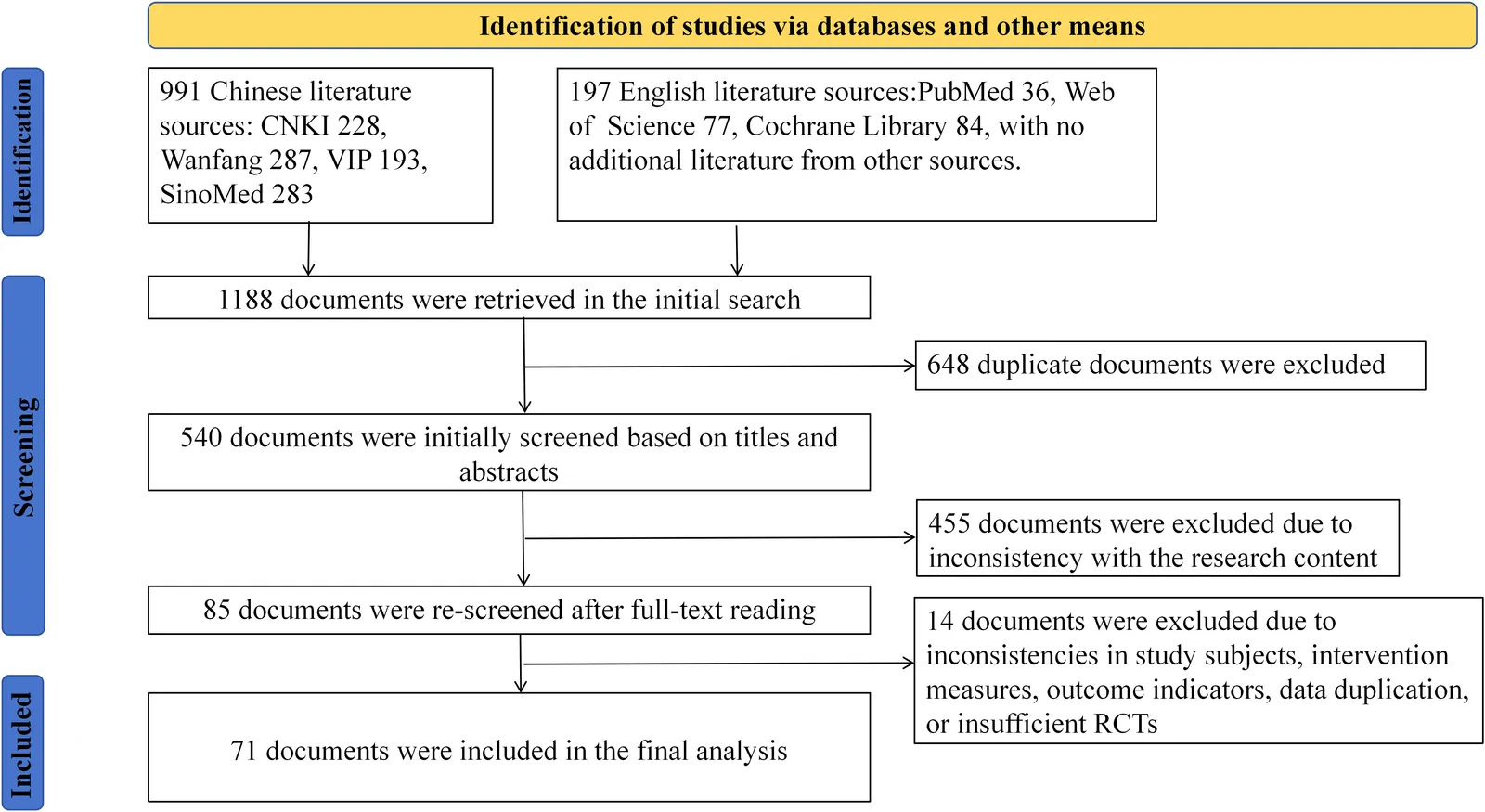
Literature screening process.

A total of 1,188 studies were retrieved from the above databases according to the search strategy, including 991 Chinese-language articles and 197 English-language articles. Based on the inclusion and exclusion criteria, 1,117 studies were excluded, and a total of 71 studies (11–81) were finally included, all of which were Chinese-language publications (**Figure 2**).

### 3.2 Basic characteristics of included studies

A total of 71 studies (11–81) were included, all of which were two-arm trials, involving 6,386 patients in total, with 3,203 patients in the experimental group and 3,183 patients in the control group. A total of 10 types of oral Chinese patent medicines were involved, including Kanglaite Injection (KLTI) in 15 studies (11–25); Kang’ai Injection (KAI) in 11 studies (26–36); Shenqi Fuzheng Injection (SQFZI) (37–46) and Aidi Injection (ADI) (47–56) in 10 studies each;and Fufang Kushen Injection (FFKSI) in 8 studies (57–64); Shenmai Injection (SMI) (65–69) and Yangdanzi Youru Injection (YDZYRI) (70–74) in 5 studies each; and Xiaoaiping Injection (XAPI)in 3 studies (75–77); Renshen Duotang Injection (RSDTI) (78, 79) and Huangqi Duotang Injection (HQDTI) (80, 81) in 2 studies (**Table 1**).

**Table 1:** Basic characteristics of included literature.

First author and year	Sample size (n)		Age (years)		TNM clinical staging		Treatment group intervention	Control group intervention	Treatment course	Outcome measures
	T	C	T	C	T	C				
CHEN XM et al., 2024 (11)	46	46	51.06 ± 3.51	51.20 ± 3.52	IIIB-IV	IIIB-IV	KLTI+CG	DP	6 weeks	①②③④⑤⑥⑰⑱
ZHU YT, 2022 (12)	20	20	50.11 ± 2.22	50.58 ± 2.31	intermediate and advanced stages	intermediate and advanced stages	KLTI+CG	TP	3 weeks	①②④⑥⑱
ZHANG XX, 2022 (13)	68	59	–	–	III-IV	III-IV	KLTI+CG	TP/GP	6–8 weeks	⑱
YIN YL et al., 2024 (14)	30	30	63.58 ± 7.41	63.64 ± 7.38	III-IV	III-IV	KLTI+CG	PP	12 weeks	①②⑧⑨⑩⑪⑬⑭⑱
LUO X et al., 2022 (15)	40	40	59.04 ± 5.93	58.38 ± 6.72	III-IV	III-IV	KLTI+CG	GP	12 weeks	①②③④⑤⑥⑰⑱
CHEN HL et al., 2023 (16)	40	40	65.52 ± 5.18	65.18 ± 5.37	III-IV	III-IV	KLTI+CG	GP	6 weeks	⑰
YANG LX et al., 2021 (17)	45	45	59.4 ± 6.9	60.7 ± 6.3	IIIA-IIIC	IIIA-IIIC	KLTI+CG	TP	6 weeks	①②③④⑤⑥⑪⑫⑯⑱
GUO HR et al., 2021 (18)	40	40	61.36 ± 11.27	60.51 ± 10.94	III-IV	III-IV	KLTI+CG	DP	6 weeks	①②⑫⑮⑱
ZHOU J et al., 2021 (19)	40	40	61.58 ± 8.43	62.01 ± 8.27	III-IV	III-IV	KLTI+CG	DP	6 weeks	①②⑪⑬⑭
CHEN QT, 2022 (20)	26	26	60.74 ± 4.11	59.67 ± 4.43	advanced stage	advanced stage	KLTI+CG	GP	3 weeks	①②⑱
LIU Y, 2021 (21)	14	16	–	–	III-IV	III-IV	KLTI+CG	GP/TP	12 weeks	①②⑪⑫⑱
GE Y et al., 2025 (22)	28	28	59.14 ± 2.26	59.12 ± 2.33	advanced stage	advanced stage	KLTI+CG	GP	3 weeks	③④⑤⑥⑱
ZHANG KS et al., 2021 (23)	48	48	62.0 ± 5.7	62.3 ± 5.5	III-IV	III-IV	KLTI+CG	GP	3 weeks	①②⑱
GAO YJ et al., 2022 (24)	40	40	52.96 ± 4.61	53.15 ± 4.54	IV	IV	KLTI+CG	TP	12 weeks	①②③④⑤⑥⑱
ZHAN LQ et al., 2021 (25)	49	49	64.19 ± 2.56	64.51 ± 2.81	IIIB-IV	IIIB-IV	KLTI+CG	GP	6 weeks	①②③④⑤⑥
WU XY et al., 2022 (26)	50	50	66.08 ± 4.03	65.17 ± 3.20	III-IV	III-IV	KAI+CG	TP	6 weeks	①②③④⑤⑥⑰⑱
CHEN X et al., 2021 (27)	69	69	61.85 ± 7.35	60.58 ± 8.23	IIIA-IV	IIIA-IV	KAI+CG	TP	6 weeks	①②⑰
CHENG ZH, 2022 (28)	44	44	57.13 + 6.15	56.89 + 5.94	IIIB-IV	IIIB-IV	KAI+CG	GP	6 weeks	①②③④⑤⑥⑦⑱
NIE X et al., 2025 (29)	44	44	60.86 ± 6.27	60.41 ± 6.18	III-IV	III-IV	KAI+CG	TP	9 weeks	①②④⑤⑥⑪⑫⑬⑭⑱
LIU XL, 2022 (30)	75	75	66.43 ± 3.87	65.92 ± 3.26	IIIB-IV	IIIB-IV	KAI+CG	Docetaxel/Gemcitabine	4 weeks	⑪⑰⑱
WANG ZH et al., 2024 (31)	55	55	68.56 ± 3.77	68.45 ± 3.81	advanced stage	advanced stage	KAI+CG	TP	6 weeks	①②⑪⑰⑱
ZHANG SC et al., 2021 (32)	45	45	68.3 ± 6.7	68.7 ± 6.3	IIIA-IV	IIIA-IV	KAI+CG	TP	6 weeks	①②⑱
HUANG Z et al., 2021 (33)	49	45	67.45 ± 4.94	68.04 ± 5.28	IIIB-IV	IIIB-IV	KAI+CG	GP	12 weeks	①②⑱
LIU XY et al., 2021 (34)	34	34	–	–	advanced stage	advanced stage	KAI+CG	GP	3 weeks	③④⑤⑪⑫
WU YQ, 2021 (35)	37	35	62.6 ± 5.7	62.8 ± 5.5	IIIB-IV	IIIB-IV	KAI+CG	GP	8 weeks	①②③④⑤⑥⑦⑱
GAO ZH et al., 2021 (36)	27	27	56.62 ± 7.59	55.74 ± 8.63	advanced stage	advanced stage	KAI+CG	GP	40 days	①②⑱
HE WX, 2021 (37)	48	48	67.03 ± 4.64	65.94 ± 4.85	III-IV	III-IV	SQFZI+CG	GP	12 weeks	①②③④⑥⑪⑫⑬⑮⑱
WANG HL, 2021 (38)	53	53	59.54 ± 6.21	60.51 ± 6.22	IIIB-IV	IIIB-IV	SQFZI+CG	GP	6 weeks	①②③④⑤⑥⑦⑱
LIU YF et al., 2021 (39)	34	34	65.83 ± 5.34	64.25 ± 5.46	III-IV	III-IV	SQFZI+CG	GP	6 weeks	①②③④⑥
SHANG SP et al., 2022 (40)	43	43	59.22 ± 3.47	58.76 ± 3.63	IIIA-IIIB	IIIA-IIIB	SQFZI+CG	NP	6 weeks	①②③④⑤⑥⑧⑨⑩
ZHANG HH et al., 2023 (41)	34	34	47.25 ± 5.16	51.28 ± 5.88	IV	IV	SQFZI+CG	PP	12 weeks	⑪⑱
SUN Y, 2025 (42)	42	42	52.69 ± 5.87	53.49 ± 4.75	IIIB-IV	IIIB-IV	SQFZI+CG	PP	6 weeks	①②⑪⑭⑮⑱
ZHAI X et al., 2025 (43)	56	56	61.87 ± 4.35	62.15 ± 4.26	advanced stage	advanced stage	SQFZI+CG	TP/PP	12 weeks	①②③⑤⑮⑪⑫⑬⑱
SU JW et al., 2024 (44)	66	65	63.28 ± 6.91	63.76 ± 7.22	IIIB-IV	IIIB-IV	SQFZI+CG	PP	12 weeks	①②⑪⑫⑱
LEI XP et al., 2023 (45)	54	54	63.37 ± 5.02	62.56 ± 5.10	III-IV	III-IV	SQFZI+CG	NP	40 days	①②⑱
WANG JK et al., 2024 (46)	120	120	63.48 ± 8.17	59.68 ± 9.56	III-IV	III-IV	SQFZI+CG	TP	6 weeks	⑱
XIONG XF et al., 2021 (47)	48	48	72.16 ± 2.86	71.48 ± 3.15	IIIB-IV	IIIB-IV	ADI+CG	TP	–	①②③④⑤⑥⑰⑱
LIANG HM et al., 2023 (48)	60	60	67.24 ± 5.92	66.89 ± 6.38	IIIB-IV	IIIB-IV	ADI+CG	GP	6 weeks	①②③④⑤
LIAN SH et al., 2024 (49)	31	31	67.58 ± 3.29	67.55 ± 2.78	IIIB-IV	IIIB-IV	ADI+CG	GP	6 weeks	①②③④⑤
YANG J et al., 2024 (50)	48	48	58.45 ± 2.25	58.57 ± 2.40	III-IV	III-IV	ADI+CG	DP	6 weeks	①②⑪⑫⑱
HUANG LS et al., 2023 (51)	36	36	61.85 ± 4.39	61.74 ± 4.47	intermediate and advanced stages	intermediate and advanced stages	ADI+CG	TP	6 weeks	①②⑪⑫⑰⑱
FU HZ et al., 2022 (52)	38	38	58.72 ± 3.67	58.69 ± 3.73	III-IV	III-IV	ADI+CG	DP	16 weeks	①②⑱
WANG F et al., 2023 (53)	44	45	52.3 ± 7.3		advanced stage	advanced stage	ADI+CG	TP	6–12 weeks	①②⑧⑨⑩⑱
XU DM, 2021 (54)	55	50	58.83 ± 7.34	59.16 ± 7.52	III-IV	III-IV	ADI+CG	GP/TP	6 weeks	①②⑱
SHEN RR et al., 2021 (55)	30	30	58.47 ± 5.95	57.41 ± 5.83	III-IV	III-IV	ADI+CG	GP	12 weeks	①②⑧⑨⑩⑱
MAO GF et al., 2021 (56)	61	61	49.81 ± 7.17	50.72 ± 6.45	III-IV	III-IV	ADI+CG	PP	6–12 weeks	①②⑪⑫⑬⑮⑱
ZUO N et al., 2021 (57)	25	25	56.81 ± 1.27	56.75 ± 1.25	intermediate and advanced stages	intermediate and advanced stages	FFKSI+CG	PP	6 weeks	①②④⑤⑥
FAN FR et al., 2021 (58)	31	32	–	–	IIIB-IV	IIIB-IV	FFKSI+CG	DP	12 weeks	①②⑱
FAN XC et al., 2021 (59)	59	59	62.74 ± 4.32	63.67 ± 4.62	advanced stage	advanced stage	FFKSI+CG	DP	9 weeks	⑱
LIU C et al., 2022 (60)	49	49	56.29 ± 6.46	53.51 ± 5.67	advanced stage	advanced stage	FFKSI+CG	TP	6 weeks	①②④⑤⑥⑱
SONG WJ, 2021 (61)	48	48	50.21 ± 4.83	51.86 ± 5.76	advanced stage	advanced stage	FFKSI+CG	TP	6 weeks	①②⑱
ZHANG YP et al., 2023 (62)	41	41	61.8 ± 1.6	62.1 ± 1.2	III-IV	III-IV	FFKSI+CG	GP	6 weeks	①②③④⑤⑥⑪⑫⑬⑱
FENG FK et al., 2023 (63)	34	34	58.47 ± 7.16	58.88 ± 7.58	IV	IV	FFKSI+CG	Cisplatin	3 weeks	①②⑪⑰⑱
YOU J, 2023 (64)	45	45	62.39	63.87	IIIA-IIIC	IIIA-IIIC	FFKSI+CG	TP	6 weeks	①②④⑤⑥⑱
XU SS et al., 2025 (65)	50	50	55.73 ± 5.44	55.39 ± 5.63	advanced stage	advanced stage	SMI+CG	GP	12 weeks	①②⑱
XU L et al., 2021 (66)	63	63	60.27 ± 8.43	60.31 ± 8.29	IIIA-IV	IIIA-IV	SMI+CG	PP	4 weeks	①②③④⑤⑱
CAI J et al., 2023 (67)	40	40	69.48 ± 9.03	68.77 ± 9.14	IIIA-IV	IIIA-IV	SMI+CG	TP	6 weeks	①②⑪⑫⑬⑭⑱
KUANG RQ, 2022 (68)	35	35	68.26 ± 9.03	68.61 ± 8.32	III-IV	III-IV	SMI+CG	TP/GP/DP/PP/NP	–	①②⑰⑱
CHEN HY et al., 2024 (69)	59	59	56.12 ± 6.38	56.49 ± 7.31	IIIB-IV	IIIB-IV	SMI+CG	GP	16 weeks	③④⑤⑥⑪⑫⑭⑯
LIU ZH et al., 2022 (70)	40	40	62.18 ± 4.90	62.02 ± 4.71	IIIA-IV	IIIA-IV	YDZYRI+CG	PP	9 weeks	①②⑫⑱
ZHANG Q et al., 2026 (71)	40	40	62.02 ± 8.01	60.75 ± 7.96	IIIB	IIIB	YDZYRI+CG	TP	6 weeks	①②⑪⑫⑭⑱
FANG HH et al., 2024 (72)	50	50	58.16 ± 5.84	58.72 ± 6.19	III-IV	III-IV	YDZYRI+CG	TP	12 weeks	①②⑪⑫⑯⑱
GE XX, 2023 (73)	36	36	57.29 ± 8.27	56.85 ± 9.15	advanced stage	advanced stage	YDZYRI+CG	NP	9 weeks	①②③④⑤⑥
WANG J et al., 2024 (74)	48	48	54.61 ± 5.67	54.78 ± 5.49	intermediate and advanced stages	intermediate and advanced stages	YDZYRI+CG	GP	9 weeks	①②③④⑤⑥⑱
MING H et al., 2024 (75)	30	30	65.52 ± 2.24	65.67 ± 2.21	IIIB-IV	IIIB-IV	XAPI+CG	GP	9 weeks	①②③④⑥⑱
YANG R et al., 2025 (76)	39	39	64.31 ± 7.42	63.77 ± 6.17	III-IV	III-IV	XAPI+CG	PP	18 weeks	①②⑪⑫⑱
LIU DL et al., 2023 (77)	53	53	63.73 ± 5.43	60.07 ± 5.84	IIIB-IV	IIIB-IV	XAPI+CG	TP	4 weeks	①②⑫⑯⑱
WANG J et al., 2022 (78)	24	24	58.31 ± 2.59	58.26 ± 2.63	advanced stage	advanced stage	RSDTI+CG	PP	12–16 weeks	①②③④⑤⑪⑫
WANG M et al., 2022 (79)	40	40	59.84 ± 7.46	59.36 ± 6.28	IIIB-IV	IIIB-IV	RSDTI+CG	DP	12 weeks	①②⑪⑫⑮⑱
SUN CZ, 2025 (80)	58	55	60.98 ± 7.02	61.45 ± 6.85	IV	IV	HQDTI+CG	PP	6 weeks	①②⑪⑫⑱
WANG SF et al., 2022 (81)	62	62	60.87 ± 6.92	61.59 ± 6.43	advanced stage	advanced stage	HQDTI+CG	PP	12 weeks	①②③④⑥⑪⑫⑱

Intervention strategy: DP: Docetaxel plus cisplatin regimen; TP: Paclitaxel plus platinum regimen; AP: Pemetrexed plus cisplatin regimen; GP: Gemcitabine plus platinum regimen; NP: Vinorelbine plus platinum regimen; ①ORR or CR + PR; ②DCR or CR + PR + SD; ③CD3 ^+^T; ④CD4 ^+^T; ⑤CD8 ^+^T; ⑥CD4 ^+^T/CD8 ^+^T ratio; ⑦NK Cell; ⑧IgA; ⑨IgG; ⑩IgM; ⑪CEA; ⑫CYFRA21-1; ⑬NSE; ⑭CA125; ⑮CA19-9; ⑯SCC; ⑰VEGF; ⑱Adverse reactions; “-” indicates not mentioned.

### 3.3 Risk of bias assessment for included studies

Among the 71 included randomized controlled trials (RCTs), 46 (15, 17, 18, 22–33, 36–39, 42, 44, 45, 47, 50, 52–57, 59–63, 66, 68–74, 79–81) adopted the random number table method. One trial each used single-blind drawing (16), computerized randomization (43), random draw (14), random ball drawing (78), coin flipping (49), and minimization (13), all of which were rated as “low risk”. The remaining trials only mentioned randomization without specifying the method, and were categorized as “unclear risk”. All included studies failed to describe allocation concealment and blinding, which were thus rated as “unclear risk”. Two studies (17, 21) reported participant dropouts, exclusions and corresponding handling measures, with complete data, and were assessed as “low risk” for incomplete outcome data. Regarding selective reporting, none of the trials were registered in the China Clinical Trial Registry and no study protocols could be retrieved, leading to “unclear risk”. No other sources of bias were reported in all literatures, which were therefore judged as “unclear risk”.

**Figure 3:**
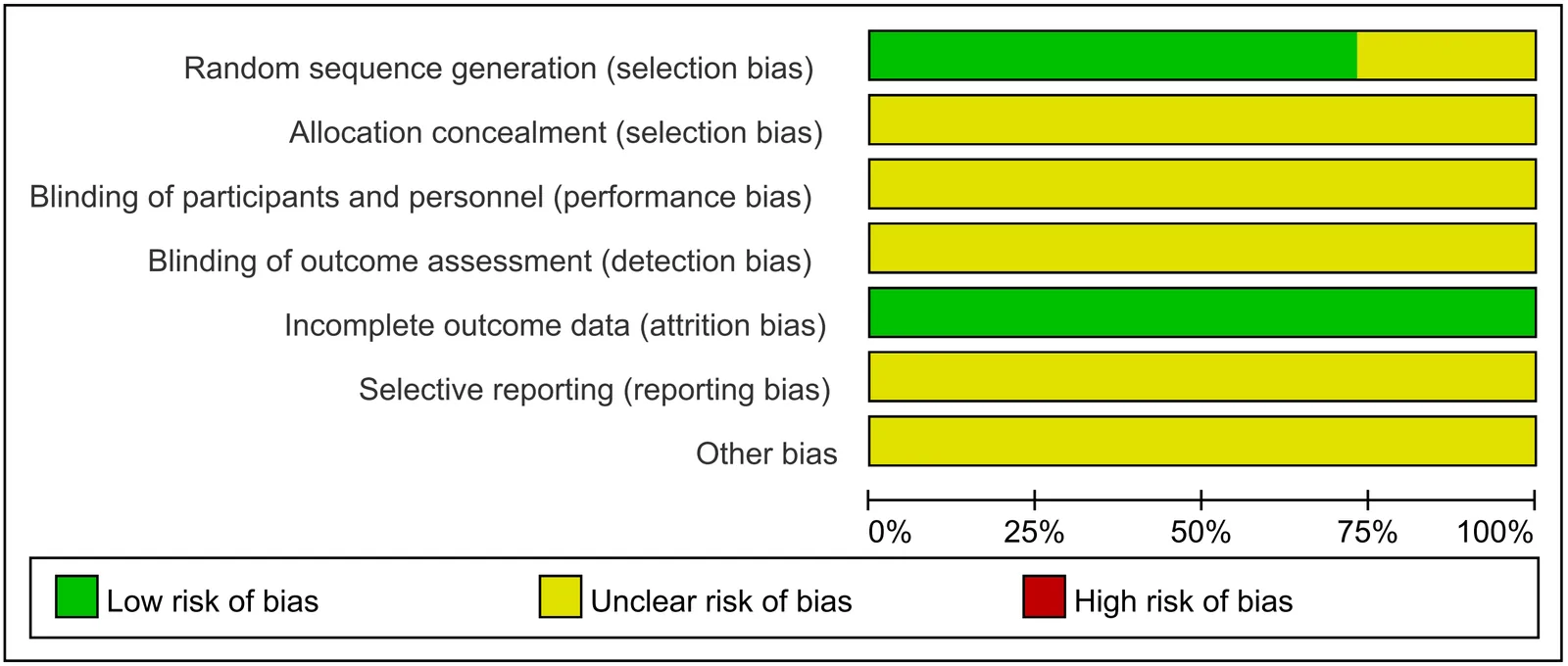
Risk of bias of the included studies.

Overall, the included studies exhibited substantial methodological limitations in the randomization process, allocation concealment, blinding, and selective reporting. Specifically, none of the included trials reported allocation concealment or blinding procedures, and no prospectively registered study protocols were available. These deficiencies raised serious concerns regarding selection bias, performance bias, detection bias, and selective reporting bias, resulting in a high overall risk of bias across the evidence base (**Figure 3**).

### 3.4 Network plot of evidence

**Figure 4:**
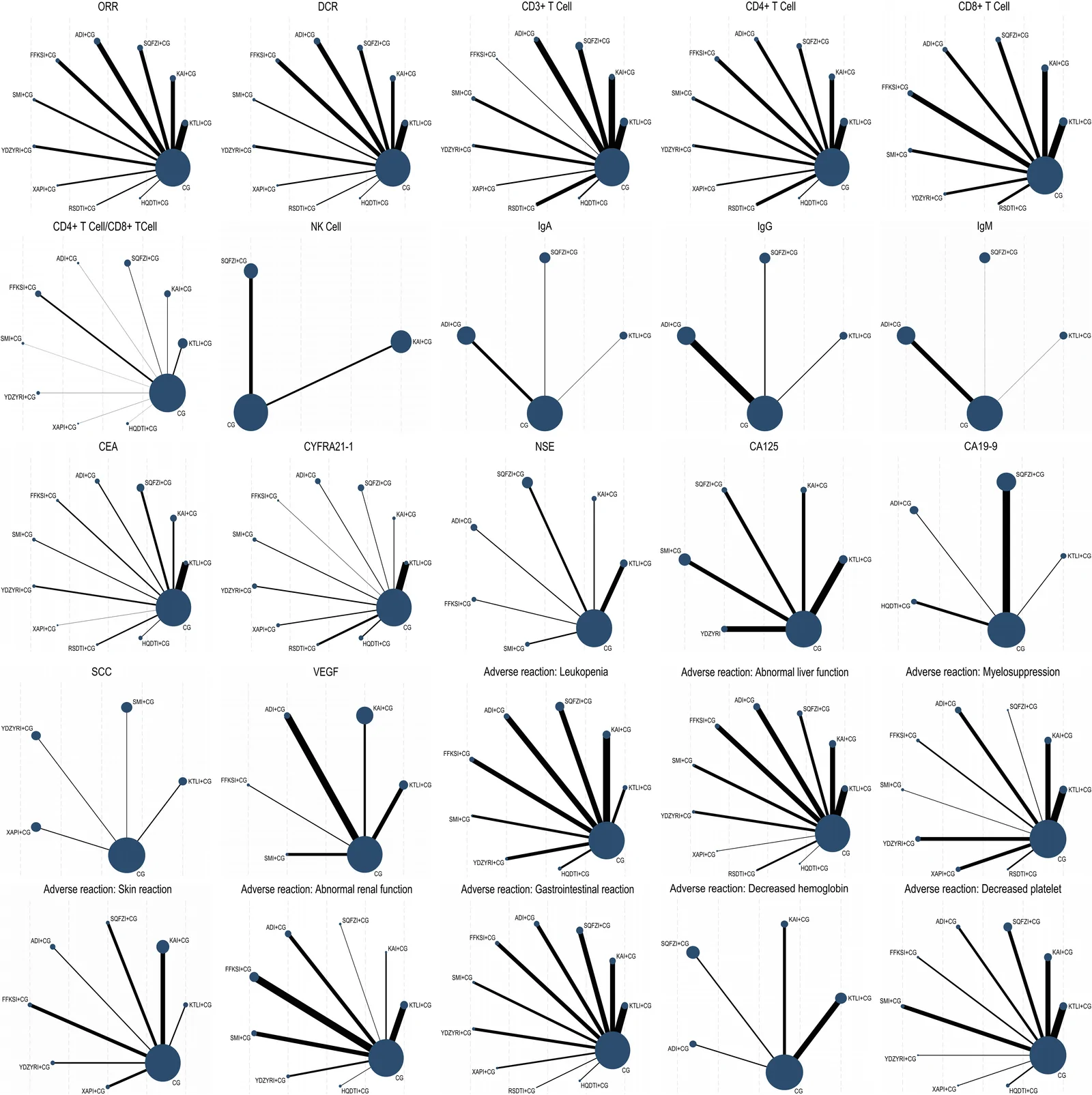
Network evidence plots for all outcome indicators.

This study included 10 TCMIs for the treatment of NSCLC and constructed network evidence plots (**Figure 4**) based on all outcome indicators. Each node in the plots stands for a distinct intervention regimen, and the node size varies with the sample size - a larger node indicates a greater number of enrolled patients for the corresponding intervention. The connecting lines represent direct comparisons between different interventions; the line thickness is determined by the number of included controlled studies, and thicker lines signify more studies comparing the two interventions.

### 3.5 Results of network meta-analysis

#### 3.5.1 Inconsistency test

As shown in the evidence network plots for all outcome indicators (**Figure 4**), none of the 10 included TCMIs regimens had head-to-head direct comparative trials, and all interventions were only connected to chemotherapy alone, forming an entirely star-shaped network without closed loops for node-splitting inconsistency test. Hence, quantitative inconsistency assessment could not be conducted. Comparisons among various TCMIs were only realized via indirect inference with chemotherapy alone as the common comparator, lacking direct clinical trial data to verify the consistency of indirect findings. Given the network characteristic, the consistent Bayesian model was adopted for pooled analysis. Potential inconsistency bias on pooled effect sizes and SUCRA rankings could not be ruled out, so the ranking results derived from indirect comparison should be interpreted prudently in clinical application.

#### 3.5.2 Evaluation of model convergence

Bayesian NMA was performed in R 4.3.1 with gemtc and netmeta packages based on MCMC algorithm. Three Markov chains with disparate initial parameters were set, including 1000 adaptive iterations, 1000 burn-in iterations and total 10000 iterations with thinning interval of 1; effective posterior sample size was 9000 after excluding burn-in samples. Convergence was judged by PSRF. Calculation results demonstrated that PSRF values of all endpoints including objective response rate, disease control rate, immune indexes, tumor markers and adverse reaction rates ranged from 1.00 to 1.05 (all below the cutoff value of 1.1). The iteration curves of three chains overlapped well without drastic fluctuation, indicating favorable model convergence and stable parameter estimation. Accordingly, the dataset was statistically eligible for subsequent pooled effect calculation and SUCRA ranking.

#### 3.5.3 ORR

**Figure 5:**
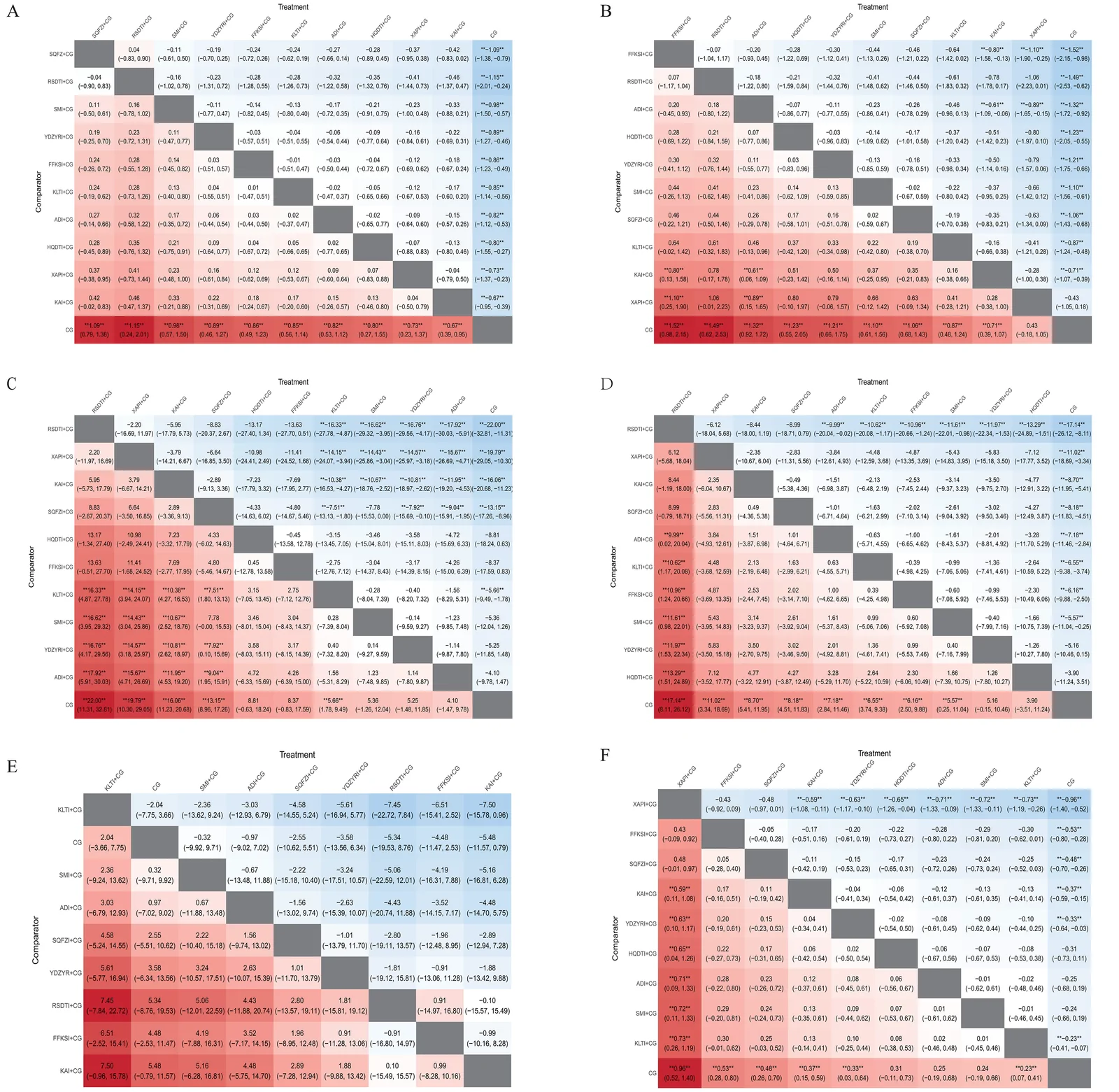
Heatmaps of ORR, DCR and immune function indicators. **(A)** ORR; **(B)** DCR; **(C)** CD3^+^ T Cell; **(D)** CD4^+^ T Cell; **(E)** CD8^+^ T Cell; **(F)** CD4^+^ T Cell/CD8^+^ T Cell. ** indicates a statistically significant difference between the two interventions.

A total of 62 studies (11, 12, 14, 15, 17–21, 23–29, 31–33, 35–40, 42–45, 47–58, 60–68, 70–81) with 5,361 participants were included. A total of 10 TCMIs were included. There was low statistical heterogeneity across the included studies (I^2^ = 0, P = 0.9992) (**Supplementary Figure 2A**). The results indicated that the combination of traditional Chinese medicine injections and chemotherapy produced a statistically significant improvement in patients’ objective response rate (ORR) compared with chemotherapy alone. No statistically significant differences were found in pairwise comparisons among different traditional Chinese medicine injections (**Figure 5A**).

**Figure 6:**
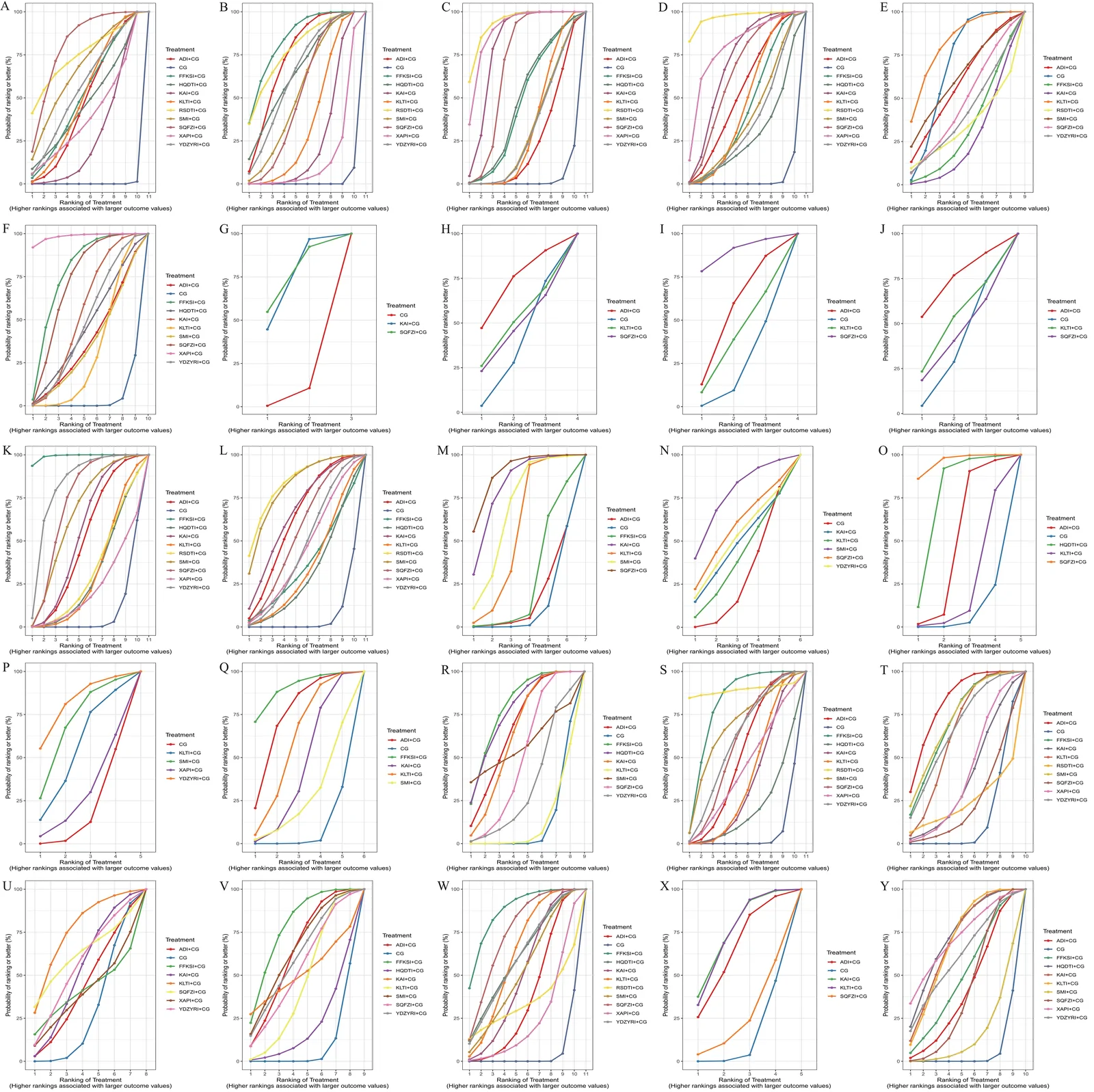
Cumulative Probability Ranking of TCMIs+CG for the Treatment of NSCLC **(A)** ORR; **(B)** DCR; **(C)** CD3^+^ T Cell; **(D)** CD4^+^ T Cell; **(E)** CD8^+^ T Cell; **(F)** CD4^+^ T Cell/CD8^+^ T Cell; **(G)** NK Cell; **(H)** IgA; **(I)** IgG; **(J)** IgM; **(K)** CEA; **(L)** CYFRA21-1; **(M)** NSE; **(N)** CA125; **(O)** CA19-9; **(P)** SCC; **(Q)** VEGF; **(R)** Adverse reaction: Leukopenia; **(S)** Adverse reaction: Abnormal liver function; **(T)** Adverse reaction: Myelosuppression; **(U)** Adverse reaction: Skin reaction; **(V)** Adverse reaction: Abnormal renal function; **(W)** Adverse reaction: Gastrointestinal reaction; **(X)** Adverse reaction: Decreased hemoglobin; **(Y)** Adverse reaction: Decreased platelet.

The SUCRA values ranked in descending order are presented as follows:

SQFZI combined with chemotherapy (SUCRA = 80.7) > RSDTI combined with chemotherapy (SUCRA = 76.2) > SMI combined with chemotherapy (SUCRA = 70.2) > YDZYRI combined with chemotherapy (SUCRA = 53.8) > KLTI combined with chemotherapy (SUCRA = 53.1) > FFKSI combined with chemotherapy (SUCRA = 53) > ADI combined with chemotherapy (SUCRA = 46.5) > HQDTI combined with chemotherapy (SUCRA = 44.4) > XAPI combined with chemotherapy (SUCRA = 41.7) > KAI combined with chemotherapy (SUCRA = 30.5) > Chemotherapy alone (SUCRA = 0) (**Figure 6A**).

#### 3.5.4 DCR

A total of 62 studies (11, 12, 14, 15, 17–21, 23–29, 31–33, 35–40, 42–45, 47–58, 60–68, 70–81) involving 5,361 participants were included. A total of 10 TCMIs were included. Low statistical heterogeneity was detected across studies (I^2^ = 14.6, P = 0.1701) (**Supplementary Figure 2B**). The results showed that all TCMIs, except XAPI, significantly improved patients’ DCR compared with chemotherapy alone. In pairwise comparisons among different injections, FFKSI combined with chemotherapy was superior to KAI plus chemotherapy and XAPI plus chemotherapy. No significant differences were observed between the remaining interventions (**Figure 5B**).

The SUCRA values in descending order were as follows: RSDTI combined with chemotherapy (SUCRA = 79.7) > FFKSI combined with chemotherapy (SUCRA = 75.4) > ADI combined with chemotherapy (SUCRA = 72.5) > HQDTI combined with chemotherapy (SUCRA = 68.7) > YDZYRI combined with chemotherapy (SUCRA = 64.3) > SMI combined with chemotherapy (SUCRA = 55.9) > SQFZI combined with chemotherapy (SUCRA = 52.8) > KLTI combined with chemotherapy (SUCRA = 38.5) > KAI combined with chemotherapy (SUCRA = 24.7) > XAPI combined with chemotherapy (SUCRA = 16.9) > Chemotherapy alone (SUCRA = 0.7) (**Figure 6B**).

#### 3.5.5 Immune function indicators

##### 3.5.5.1 CD3^+^; T cells

A total of 26 studies (11, 15, 17, 22, 24–26, 28, 34, 35, 37–40, 43, 47–49, 62, 66, 69, 73–75, 78, 81) involving 2,296 participants and 10 types of TCMIs were included. Substantial statistical heterogeneity was observed across all studies(I^2^ = 98.6,P=0) (**Supplementary Figure 2C**). The results indicated that compared with chemotherapy alone, the combination of RSDTI, XAPI, KAI or SQFZI with chemotherapy significantly elevated the level of CD3^+^; T cells. In pairwise comparisons among different TCMIs, the combined regimens of RSDTI, XAPI and KAI plus chemotherapy exhibited superior efficacy relative to KLTI, SMI, YDZYRI and ADI combined with chemotherapy. SQFZI combined with chemotherapy was more effective than KLTI, YDZYRI and ADI combined with chemotherapy. No significant differences were detected in the remaining pairwise comparisons (**Figure 5C**).

The SUCRA values were ranked in descending order as follows: RSDTI combined with chemotherapy (SUCRA = 93.6) > XAPI combined with chemotherapy (SUCRA = 90.3) > KAI combined with chemotherapy (SUCRA = 80.8) > SQFZI combined with chemotherapy (SUCRA = 69.3) > HQDTI combined with chemotherapy (SUCRA = 48.4) > FFKSI combined with chemotherapy (SUCRA = 46.8) > KLTI combined with chemotherapy (SUCRA = 33.1) > SMI combined with chemotherapy (SUCRA = 32.1) > YDZYRI combined with chemotherapy (SUCRA = 30.7) > ADI combined with chemotherapy (SUCRA = 22.8) > Chemotherapy alone (SUCRA = 1.9) (**Figure 6C**).

##### 3.5.5.2 CD4^+^; T cells

30 studies (11, 12, 15, 17, 22, 24–26, 28, 29, 34, 35, 37–40, 47–49, 57, 60, 62, 64, 66, 73–75, 78, 81) with a total of 2,550 participants and 10 types of TCMIs were included. Significant statistical heterogeneity was found across studies(I^2^ = 96,P<0.0001) (**Supplementary Figure 2D**). The results demonstrated that all TCMIs, except YDZYRI and HQDTI, significantly increased the level of CD4^+^; T cells when combined with chemotherapy compared with chemotherapy alone. In pairwise comparisons, RSDTI combined with chemotherapy yielded better efficacy than ADI, KLTI, FFKSI, SMI, YDZYRI and HQDTI combined with chemotherapy. No significant differences were observed between the other paired interventions (**Figure 5D**).

The SUCRA values in descending order were as follows: RSDTI combined with chemotherapy (SUCRA = 97) > XAPI combined with chemotherapy (SUCRA = 79.5) > KAI combined with chemotherapy (SUCRA = 69.5) > SQFZI combined with chemotherapy (SUCRA = 63.3) > ADI combined with chemotherapy (SUCRA = 53.3) > KLTI combined with chemotherapy (SUCRA = 45.6) > FFKSI combined with chemotherapy (SUCRA = 42.7) > SMI combined with chemotherapy (SUCRA = 37) > YDZYRI combined with chemotherapy (SUCRA = 34.3) > HQDTI combined with chemotherapy (SUCRA = 26.5) > Chemotherapy alone (SUCRA = 1.4) (**Figure 6D**).

##### 3.5.5.3 CD8^+^; T cells

A total of 26 studies (11, 15, 17, 22, 24–26, 28, 29, 34, 35, 38, 40, 43, 47–49, 57, 60, 62, 64, 66, 69, 73, 74, 78) involving 2,274 participants and 8 types of TCMIs were included. Considerable statistical heterogeneity was detected across studies(I^2^ = 99.3,P=0) (**Supplementary Figure 2E**). The results showed that no statistically significant differences were found in comparisons between TCMIs combined with chemotherapy and chemotherapy alone, as well as among different intervention groups (**Figure 5E**).

The surface under the cumulative ranking curve (SUCRA) values ranked in descending order were as follows: KLTI combined with chemotherapy (SUCRA = 82.3) > Chemotherapy alone (SUCRA = 70) > SMI combined with chemotherapy (SUCRA = 64) > ADI combined with chemotherapy (SUCRA = 58.2) > SQFZI combined with chemotherapy (SUCRA = 45.9) > YDZYRI combined with chemotherapy (SUCRA = 40.8) > FFKSI combined with chemotherapy (SUCRA = 32.7) > RSDTI combined with chemotherapy (SUCRA = 31.4) > KAI combined with chemotherapy (SUCRA = 24.8) (**Figure 6E**)

##### 3.5.5.4 CD4^+^; T cells/CD8^+^; T cells radio

A total of 25 studies (11, 12, 15, 17, 22, 24–26, 28, 29, 35, 37–40, 47, 57, 60, 62, 64, 69, 73–75, 81) including 2,126 participants and 9 types of TCMIs were enrolled. Substantial statistical heterogeneity was observed across studies(I^2^ = 99.3,P=0) (**Supplementary Figure 2F**). The results revealed that compared with chemotherapy alone, XAPI, FFKSI, SQFZI, KAI and YDZYRI significantly elevated the ratio of CD4^+^; T cells to CD8^+^; T cells. In pairwise comparisons of different TCMIs, XAPI combined with chemotherapy was superior to KAI, YDZYRI, HQDTI, ADI, SMI and KLTI combined with chemotherapy. No statistically significant differences were identified in the remaining pairwise comparisons (**Figure 5F**).

The SUCRA values in descending order were listed as follows: XAPI combined with chemotherapy (SUCRA = 99.2) > FFKSI combined with chemotherapy (SUCRA = 78.1) > SQFZI combined with chemotherapy (SUCRA = 72.8) > KAI combined with chemotherapy (SUCRA = 53.9) > YDZYRI combined with chemotherapy (SUCRA = 46.6) > HQDTI combined with chemotherapy (SUCRA = 44.4) > ADI combined with chemotherapy (SUCRA = 35.5) > SMI combined with chemotherapy (SUCRA = 35.2) > KLTI combined with chemotherapy (SUCRA = 31.1) > chemotherapy alone (SUCRA = 3.1). (**Figure 6F**)

##### 3.5.5.5 NK Cell

**Figure 7:**
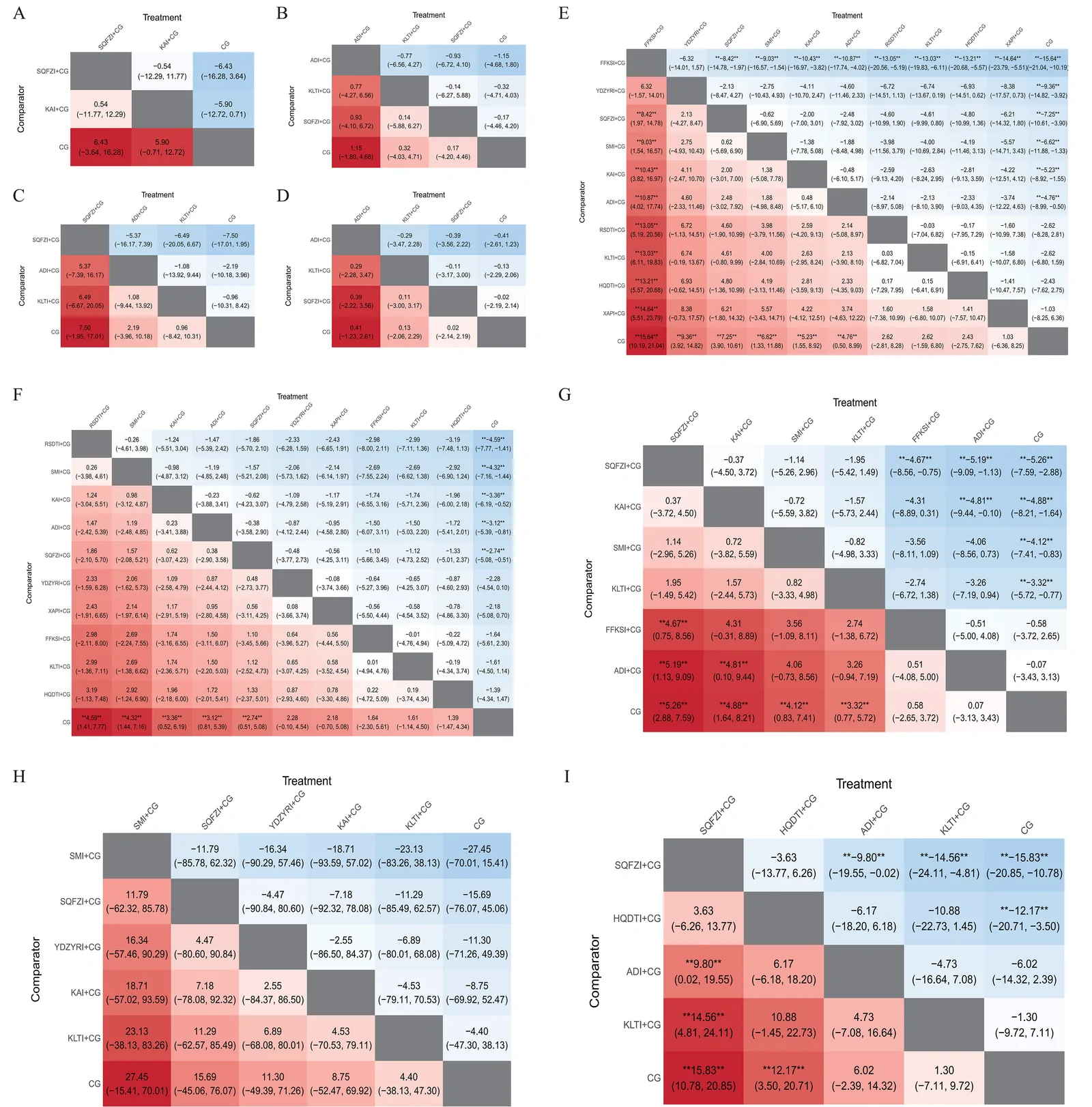
Heatmaps of NK cell, immunoglobulin indicators and tumor marker indicators **(A)** NK Cell; **(B)** IgA; **(C)** IgG; **(D)** IgM; **(E)** CEA; **(F)** CYFRA21-1; **(G)** NSE; **(H)** CA125; **(I)** CA19-9. ** indicates a statistically significant difference between the two interventions.

A total of 3 studies (28, 35, 38) involving 266 participants and 2 types of TCMIs were included. High statistical heterogeneity was detected across studies(I^2^ = 92,P<0.0001) (**Supplementary Figure 2G**). The results indicated that there were no statistically significant differences between TCMIs combined with chemotherapy and chemotherapy alone, nor among different intervention groups (**Figure 7A**). The SUCRA values in descending order were as follows: SQFZI combined with chemotherapy (SUCRA = 77.9) > KAI combined with chemotherapy (SUCRA = 71.6) > chemotherapy alone (SUCRA = 0.4). (**Figure 6G**)

#### 3.5.6 Immunoglobulin indicators

##### 3.5.6.1 IgA

A total of 5 studies (14, 40, 41, 53, 55) with 363 participants and 3 types of TCMIs were included. Moderate to high statistical heterogeneity was observed across studies(I^2^ = 72.7,P=0.0117) (**Supplementary Figure 2H**). The results showed no statistically significant differences between TCMIs combined with chemotherapy and chemotherapy alone, as well as among different intervention groups (**Figure 7B**). The SUCRA values ranked in descending order were as follows: ADI combined with chemotherapy (SUCRA = 71.2) > KLTI combined with chemotherapy (SUCRA = 48.8) > SQFZI combined with chemotherapy (SUCRA = 44.5) > chemotherapy alone (SUCRA = 35.6). (**Figure 6H**)

##### 3.5.6.2 IgG

A total of 4 studies (14, 40, 53, 55) involving 295 participants and 3 types of TCMIs were included. High statistical heterogeneity was found across studies (I^2^ = 97.8,P<0.0001) (**Supplementary Figure 2I**). The results indicated no statistically significant differences between TCMIs combined with chemotherapy and chemotherapy alone, or among different interventions (**Figure 7C**). The SUCRA values ranked in descending order were as follows: SQFZI combined with chemotherapy (SUCRA = 91.4) > ADI combined with chemotherapy (SUCRA = 53.5) > KLTI combined with chemotherapy (SUCRA = 35.4) > chemotherapy alone (SUCRA = 19.7). (**Figure 6I**)

##### 3.5.6.3 IgM

A total of 4 (14, 40, 53, 55) studies involving 295 participants and 3 types of TCMIs were included. High statistical heterogeneity was observed across studies (I^2^ = 95.8,P<0.0001) (**Supplementary Figure 2J**). The results showed no statistically significant differences between TCMIs combined with chemotherapy and chemotherapy alone, or among different interventions (**Figure 7D**). The SUCRA values ranked in descending order were as follows: ADI combined with chemotherapy (SUCRA = 99.6) > KLTI combined with chemotherapy (SUCRA = 60.1) > SQFZI combined with chemotherapy (SUCRA = 27.8) > chemotherapy alone (SUCRA = 12.5). (**Figure 6J**)

#### 3.5.7 Tumor marker indicators

##### 3.5.7.1 CEA

A total of 27 studies (14, 17, 19, 21, 29–31, 34–37, 41–44, 50, 51, 56, 62, 63, 67, 69, 71, 72, 76, 78–81) involving 2,428 participants and 10 types of TCMIs were included. High statistical heterogeneity was detected across studiesv(I^2^ = 98.2,P<0.0001) (**Supplementary Figure 2K**). The results demonstrated that FFKSI, YDZYRI, SQFZI, SMI, KAI and ADI significantly reduced CEA levels compared with chemotherapy alone. In pairwise comparisons, FFKSI combined with chemotherapy was superior to all other TCMI regimens except YDZYRI combined with chemotherapy. No significant differences were found between the remaining interventions (**Figure 7E**).

The SUCRA values ranked in descending order were as follows: FFKSI combined with chemotherapy (SUCRA = 99.6) > YDZYRI combined with chemotherapy (SUCRA = 83.5) > SQFZI combined with chemotherapy (SUCRA = 72.8) > SMI combined with chemotherapy (SUCRA = 66.5) > KAI combined with chemotherapy (SUCRA = 54.9) > ADI combined with chemotherapy (SUCRA = 50.6) > KLTI combined with chemotherapy (SUCRA = 31.7) = RSDTI combined with chemotherapy (SUCRA = 31.7) > HQDYI combined with chemotherapy (SUCRA = 29.6) > XAPI combined with chemotherapy (SUCRA = 21.9) > chemotherapy alone (SUCRA = 8) (**Figure 6K**).

##### 3.5.7.2 CYFRA21-1

A total of 23 studies (17, 18, 21, 29, 34, 37, 43, 44, 50, 51, 56, 62, 67, 69–72, 76–81) involving 2,074 participants and 10 types of TCMIs were included. High statistical heterogeneity was observed across studies (I^2^ = 98.4,P<0.0001) (**Supplementary Figure 2L**). The results showed that RSDTI, SMI, KAI, ADI and SQFZI could significantly decrease CYFRA21–1 levels compared with chemotherapy alone. No significant differences were detected in pairwise comparisons among different TCMIs (**Figure 7F**).

The SUCRA values ranked in descending order were as follows: RSDTI combined with chemotherapy (SUCRA = 84.7) > SMI combined with chemotherapy (SUCRA = 84.5) > KAI combined with chemotherapy (SUCRA = 66.3) > ADI combined with chemotherapy (SUCRA = 63.6) > SQFZI combined with chemotherapy (SUCRA = 57) > XAPI combined with chemotherapy (SUCRA = 44.9) > YDZYRI combined with chemotherapy (SUCRA = 44.6) > FFKSI combined with chemotherapy (SUCRA = 34.9) > KLTI combined with chemotherapy (SUCRA = 33.9) = HQDTI combined with chemotherapy (SUCRA = 33.9) > chemotherapy alone (SUCRA = 4.6) (**Figure 6L**).

##### 3.5.7.3 NSE

A total of 8 studies (14, 19, 29, 37, 43, 56, 62, 67) involving 720 participants and 6 types of TCMIs were included, with high statistical heterogeneity across the studies. (I^2^ = 95.9,P<0.0001) (**Supplementary Figure 2M**). The results indicated that SQFZI, KAI, SMI and KLTI significantly reduced CA125 levels compared with chemotherapy alone. In pairwise comparisons, SQFZI combined with chemotherapy was superior to FFKSI and ADI combined with chemotherapy, and KAI combined with chemotherapy was better than ADI combined with chemotherapy. No statistically significant differences were observed between the remaining interventions (**Figure 7G**).

The SUCRA values ranked in descending order were as follows: SQFZI combined with chemotherapy (SUCRA = 94.9) > KAI combined with chemotherapy (SUCRA = 84.5) > SMI combined with chemotherapy (SUCRA = 67) > KLTI combined with chemotherapy (SUCRA = 53.6) > FFKSI combined with chemotherapy (SUCRA = 29.8) > ADI combined with chemotherapy (SUCRA = 12.2) > chemotherapy alone (SUCRA = 7.9) (**Figure 6M**).

##### 3.5.7.4 CA125

A total of 7 studies (14, 19, 29, 42, 67, 69, 71) involving 590 participants and 5 types of TCMIs were included. High statistical heterogeneity was observed across studies (I^2^ = 99.6,P<0.0001) (**Supplementary Figure 2N**). The results showed no statistically significant differences between TCMIs combined with chemotherapy and chemotherapy alone, as well as among different interventions (**Figure 7H**). The SUCRA values ranked in descending order were as follows: SMI combined with chemotherapy (SUCRA = 81.3) > SQFZI combined with chemotherapy (SUCRA = 58.5) > YDZYRI combined with chemotherapy (SUCRA = 50.4) > KAI combined with chemotherapy (SUCRA = 47.8) > KLTI combined with chemotherapy (SUCRA = 37.2) > chemotherapy alone (SUCRA = 24.6) (**Figure 6N**).

##### 3.5.7.5 CA19-9

A total of 6 studies (18, 37, 42, 43, 56, 79) involving 574 participants and 4 types of TCMIs were included. High statistical heterogeneity was detected across studies (I^2^ = 98.1,P<0.0001) (**Supplementary Figure 2O**). The results revealed that SQFZI and HQDTI significantly reduced CA19–9 levels compared with chemotherapy alone. In pairwise comparisons, SQFZI combined with chemotherapy was superior to ADI and KLTI combined with chemotherapy, while no significant differences were found between the remaining interventions (**Figure 7I**). The SUCRA values ranked in descending order were as follows: SQFZI combined with chemotherapy (SUCRA = 99.5) > HQDTI combined with chemotherapy (SUCRA = 75.5) > ADI combined with chemotherapy (SUCRA = 50) > KLTI combined with chemotherapy (SUCRA = 24.8) > chemotherapy alone (SUCRA = 0.2) (**Figure 6O**).

##### 3.5.7.6 SCC

**Figure 8:**
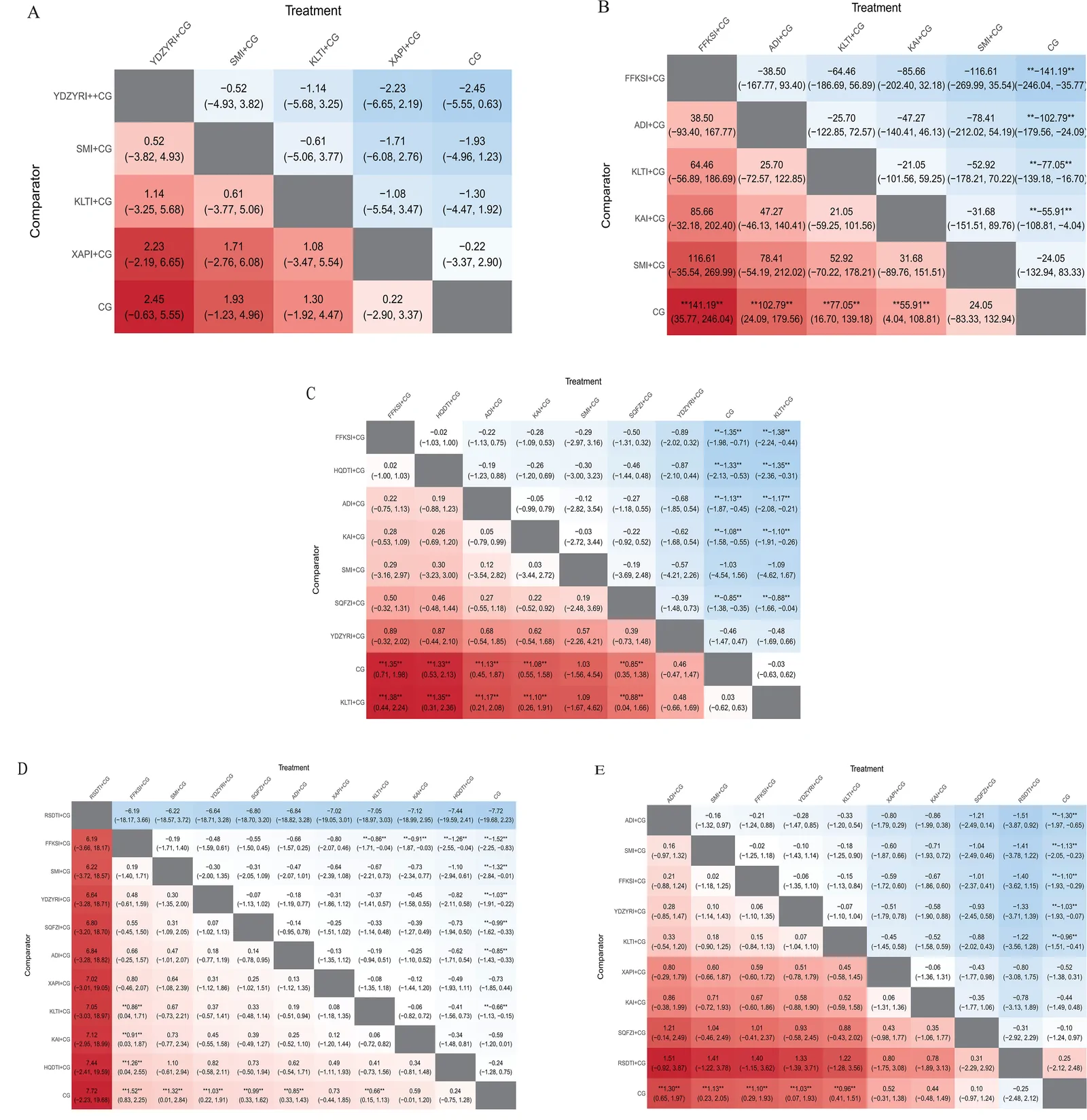
Heatmaps of SCC, VEGF and adverse reaction **(A)** SCC; **(B)** VEGF; **(C)** Adverse reaction: Leukopenia; **(D)** Adverse reaction: Abnormal liver function; **(E)** Adverse reaction: Myelosuppression. ** indicates a statistically significant difference between the two interventions.

A total of 4 studies (17, 69, 72, 77) involving 414 participants and 4 types of TCMIs were included. High statistical heterogeneity was observed across studies. (I^2^ = 88.5,P<0.0001) (**Supplementary Figure 2P**). The results showed no statistically significant differences between TCMIs combined with chemotherapy and chemotherapy alone, or among different interventions (**Figure 8A**). The SUCRA values ranked in descending order were as follows: YDZYRI combined with chemotherapy (SUCRA = 97.5) > SMI combined with chemotherapy (SUCRA = 74.2) > KLTI combined with chemotherapy (SUCRA = 52.5) > XAPI combined with chemotherapy (SUCRA = 19.2) > chemotherapy alone (SUCRA = 6.5) (**Figure 6P**).

##### 3.5.7.7 VEGF

A total of 11 studies (11, 15, 16, 26, 27, 30, 31, 47, 51, 63, 68) involving 1,156 participants and 5 types of TCMIs were included. High statistical heterogeneity was observed across studies. (I^2^ = 99,P<0.0001) (**Supplementary Figure 2Q**). The results indicated that all TCMIs except SMI significantly improved disease control rate (DCR) compared with chemotherapy alone. No significant differences were found in pairwise comparisons among different interventions (**Figure 8B**). The SUCRA values ranked in descending order were as follows: FFKSI combined with chemotherapy (SUCRA = 94.1) > ADI combined with chemotherapy (SUCRA = 75.8) > KLTI combined with chemotherapy (SUCRA = 59.1) > KAI combined with chemotherapy (SUCRA = 41) > SMI combined with chemotherapy (SUCRA = 25.9) > chemotherapy alone (SUCRA = 4.1) (**Figure 6Q**).

#### 3.5.8 Adverse reaction

##### 3.5.8.1 Leukopenia

A total of 27 studies (13, 14, 23, 26, 28, 30, 33, 35–43, 46, 50, 52, 53, 56, 58, 60–62, 67, 71, 74, 80, 81) involving 2,722 participants and 8 types of TCMIs were included. Moderate statistical heterogeneity was detected across studies. (I^2^ = 46.1,P=0.005) (**Supplementary Figure 2R**). The results showed that FFKSI, HQDTI, ADI, KAI and SQFZI exerted significant effects on alleviating leukopenia when compared with KLTI combined with chemotherapy and chemotherapy alone. No statistically significant differences were observed between the remaining interventions (**Figure 8C**).

The SUCRA values ranked in descending order were as follows: FFKSI combined with chemotherapy (SUCRA = 81.8) > XAPI combined with chemotherapy (SUCRA = 79.2) > ADI combined with chemotherapy (SUCRA = 67.9) > KAI combined with chemotherapy (SUCRA = 64.8) > SMI combined with chemotherapy (SUCRA = 50.8) > SQFZI combined with chemotherapy (SUCRA = 49.5) > YDZYRI combined with chemotherapy (SUCRA = 33) > chemotherapy alone (SUCRA = 13.4) > KLTI combined with chemotherapy (SUCRA = 9.7) (**Figure 6R**).

##### 3.5.8.2 Abnormal liver function

A total of 38 studies (12–14, 18, 21–24, 26, 30–33, 41, 42, 45–47, 51–56, 58, 60–63, 66, 68, 70, 71, 74, 77, 79, 81) involving 3,512 participants and 10 types of TCMIs were included. Low statistical heterogeneity was observed across studies (I^2^ = 0,P=0.9559) (**Supplementary Figure 2S**). The results demonstrated that RSDTI, FFKSI, SMI, YDZYRI, SQFZI, ADI and KLTI significantly relieved chemotherapy-induced liver function abnormalities compared with chemotherapy alone. In pairwise comparisons, FFKSI combined with chemotherapy was superior to KLTI, KAI and HQDTI combined with chemotherapy. No statistically significant differences were found among the other interventions (**Figure 8D**).

The SUCRA values ranked in descending order were as follows: FFKSI combined with chemotherapy (SUCRA = 85.7) > SMI combined with chemotherapy (SUCRA = 71) > YDZYRI combined with chemotherapy (SUCRA = 60.6) > RSDTI combined with chemotherapy (SUCRA = 60.3) > ADI combined with chemotherapy (SUCRA = 53.8) > XAPI combined with chemotherapy (SUCRA = 46.8) > KLTI combined with chemotherapy (SUCRA = 42.6) > KAI combined with chemotherapy (SUCRA = 37.5) > HQDTI combined with chemotherapy (SUCRA = 22.3) > chemotherapy alone (SUCRA = 7.1) (**Figure 6S**).

##### 3.5.8.3 Myelosuppression

A total of 25 studies (11, 12, 15, 18, 21, 24, 26, 28, 29, 32, 41, 47, 50, 51, 55, 63, 64, 66, 70, 72, 74–77, 79) involving 2,044 participants and 9 types of TCMIs were included. Moderate statistical heterogeneity was observed across studies (I^2^ = 40.8,P=0.0187) (**Supplementary Figure 2T**). The results showed that ADI, SMI, FFKSI, YDZYRI and KLTI significantly alleviated myelosuppression compared with chemotherapy alone. No statistically significant differences were detected in pairwise comparisons between different interventions (**Figure 8E**).

The SUCRA values ranked in descending order were as follows: ADI combined with chemotherapy (SUCRA = 83.4) > SMI combined with chemotherapy (SUCRA = 74.1) > FFKSI combined with chemotherapy (SUCRA = 70.8) > KLTI combined with chemotherapy (SUCRA = 66.5) > YDZYRI combined with chemotherapy (SUCRA = 64.6) > KAI combined with chemotherapy (SUCRA = 40.1) > XAPI combined with chemotherapy (SUCRA = 35.3) > RSDTI combined with chemotherapy (SUCRA = 28.5) > SQFZI combined with chemotherapy (SUCRA = 24) > chemotherapy alone (SUCRA = 12.8) (**Figure 6T**).

##### 3.5.8.4 Skin reaction

**Figure 9:**
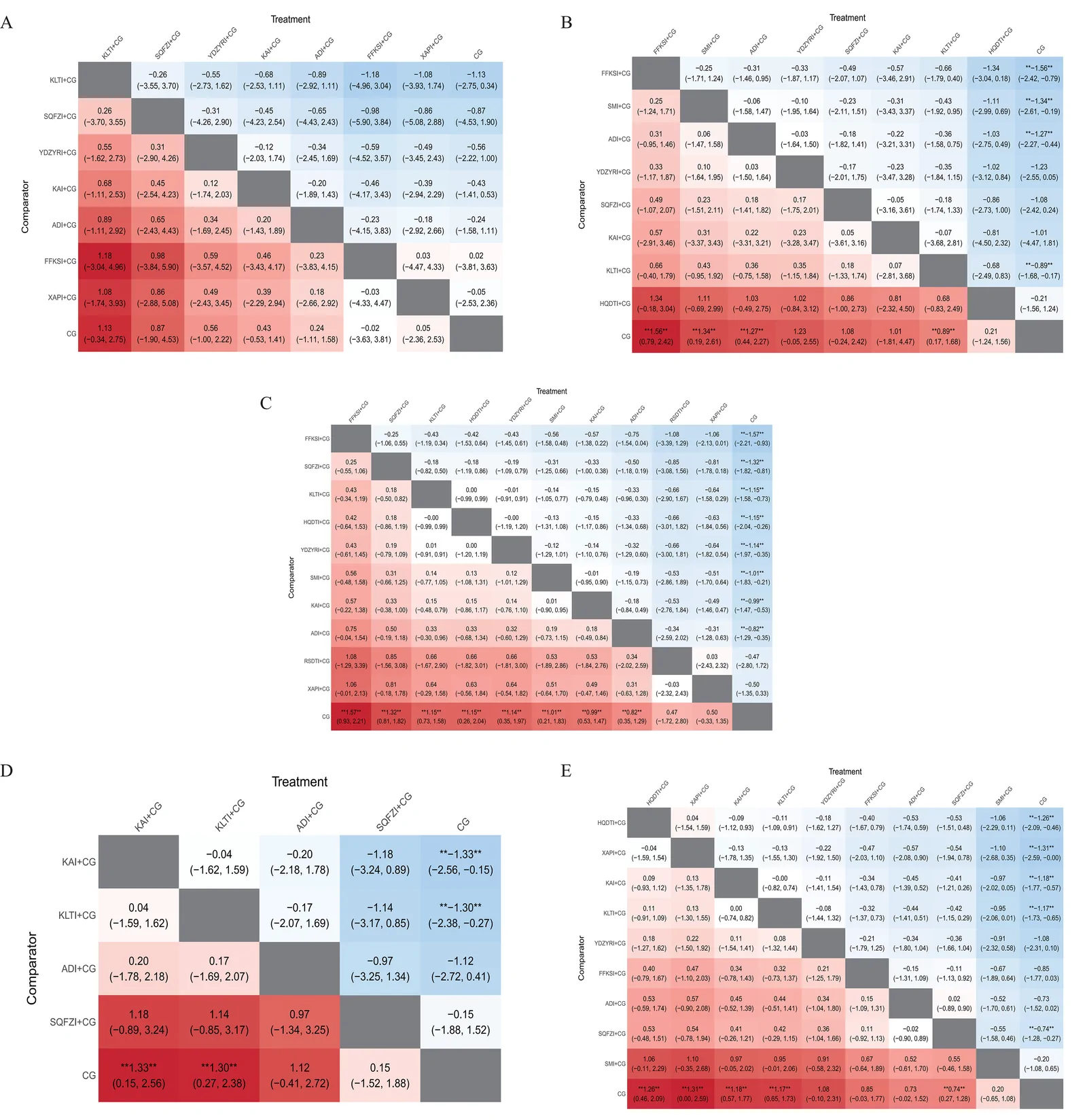
Heatmaps of adverse reaction **(A)** Adverse reaction: Skin reaction; **(B)** Adverse reaction: Abnormal renal function; **(C)** Adverse reaction: Gastrointestinal reaction; **(D)** Adverse reaction: Decreased hemoglobin; **(E)** Adverse reaction: Decreased platelet. ** indicates a statistically significant difference between the two interventions.

A total of 9 studies (11, 28, 29, 33, 41, 54, 62, 71, 76) involving 755 participants and 7 types of TCMIs were included. Low statistical heterogeneity was found across studies (I^2^ = 0,P=0.9732) (**Supplementary Figure 2U**). The results indicated no statistically significant differences between TCMIs combined with chemotherapy and chemotherapy alone, as well as among different interventions (**Figure 9A**).

The SUCRA values ranked in descending order were as follows: KLTI combined with chemotherapy (SUCRA = 80.2) > SQFZI combined with chemotherapy (SUCRA = 62.1) > YDZYRI combined with chemotherapy (SUCRA = 58.7) > KAI combined with chemotherapy (SUCRA = 53.8) > ADI combined with chemotherapy (SUCRA = 43.5) > FFKSI combined with chemotherapy (SUCRA = 38) > XAPI combined with chemotherapy (SUCRA = 36.6) > chemotherapy alone (SUCRA = 27) (**Figure 6U**).

##### 3.5.8.5 Abnormal renal function

A total of 24 studies (12, 13, 18, 21–23, 32, 38, 51, 55, 56, 58–63, 65, 66, 68, 70, 74, 81) involving 2,096 participants and 8 types of TCMIs were included. Low statistical heterogeneity was observed across studies (I^2^ = 0,P=0.6656) (**Supplementary Figure 2V**). The results showed that FFKSI, SMI, ADI and KLTI significantly relieved renal function abnormalities compared with chemotherapy alone. No statistically significant differences were found in pairwise comparisons among different interventions (**Figure 9B**).

The SUCRA values ranked in descending order were as follows: FFKSI combined with chemotherapy (SUCRA = 78.2) > SMI combined with chemotherapy (SUCRA = 64.2) > ADI combined with chemotherapy (SUCRA = 64.1) > YDZYRI combined with chemotherapy (SUCRA = 63.9) > SQFZI combined with chemotherapy (SUCRA = 58.2) > KAI combined with chemotherapy (SUCRA = 47.6) > KLTI combined with chemotherapy (SUCRA = 45.7) > HQDTI combined with chemotherapy (SUCRA = 19.4) > chemotherapy alone (SUCRA = 8.2) (**Figure 6V**).

##### 3.5.8.6 Gastrointestinal reaction

A total of 56 studies (11–15, 17, 18, 20–24, 26, 28–33, 35–37, 41–47, 50–56, 58–63, 65, 66, 68, 70–72, 74–81) involving 5,126 participants and 10 types of TCMIs were included. Low statistical heterogeneity was observed across studies (I^2^ = 27.1,P=0.0364) (**Supplementary Figure 2W**). The results showed that all TCMIs except RSDTI and XAPI significantly alleviated gastrointestinal adverse reactions compared with chemotherapy alone. No statistically significant differences were found in pairwise comparisons among different interventions (**Figure 9C**).

The SUCRA values ranked in descending order were as follows: FFKSI combined with chemotherapy (SUCRA = 85.7) > SQFZI combined with chemotherapy (SUCRA = 75.2) > KLTI combined with chemotherapy (SUCRA = 63.4) > HQDTI combined with chemotherapy (SUCRA = 63.2) > YDZYRI combined with chemotherapy (SUCRA = 61.4) > SMI combined with chemotherapy (SUCRA = 52.5) > KAI combined with chemotherapy (SUCRA = 48.9) > RSDTI combined with chemotherapy (SUCRA = 38.3) > ADI combined with chemotherapy (SUCRA = 38) > XAPI combined with chemotherapy (SUCRA = 22.9) > chemotherapy alone (SUCRA = 4.4) (**Figure 6W**).

##### 3.5.8.7 Decreased hemoglobin

A total of 7 studies (14, 20, 23, 35, 36, 46, 56) involving 696 participants and 4 types of TCMIs were included. Low statistical heterogeneity was observed across studies (I^2^ = 0,P=0.894) (**Supplementary Figure 2X**). The results indicated that KAI and KLTI significantly relieved hemoglobin reduction compared with chemotherapy alone. No statistically significant differences were found in pairwise comparisons among different interventions (**Figure 9D**).The SUCRA values ranked in descending order were as follows: KAI combined with chemotherapy (SUCRA = 78.3) > KLTI combined with chemotherapy (SUCRA = 73.7) > ADI combined with chemotherapy (SUCRA = 67.7) > ADI combined with chemotherapy (SUCRA = 19.6) > chemotherapy alone (SUCRA = 10.6) (**Figure 6X**).

##### 3.5.8.8 Decreased platelet

A total of 30 studies (13, 14, 17, 18, 20, 22, 23, 28, 30, 31, 33, 35–37, 42, 43, 45, 46, 50, 52, 56, 58, 62, 65, 67, 68, 71, 77, 80, 81) involving 2,879 participants and 9 types of TCMIs were included. High statistical heterogeneity was observed across studies (I^2^ = 0,P=0.8411) (**Supplementary Figure 2Y**). The results showed that HQDTI, XAPI, KAI, KLTI and SQFZI significantly alleviated thrombocytopenia compared with chemotherapy alone. No statistically significant differences were detected in pairwise comparisons between different interventions (**Figure 9E**).

The SUCRA values ranked in descending order were as follows: HQDTI combined with chemotherapy (SUCRA = 74.8) > KAI combined with chemotherapy (SUCRA = 70.9) > KLTI combined with chemotherapy (SUCRA = 67.3) > YDZYRI combined with chemotherapy (SUCRA = 63.1) > FFKSI combined with chemotherapy (SUCRA = 49.6) > SQFZI combined with chemotherapy (SUCRA = 42) = ADI combined with chemotherapy (SUCRA = 42) > SMI combined with chemotherapy (SUCRA = 15.2) > chemotherapy alone (SUCRA = 4.2) (**Figure 6Y**).

#### 3.5.9 Assessment of publication bias

**Figure 10:**
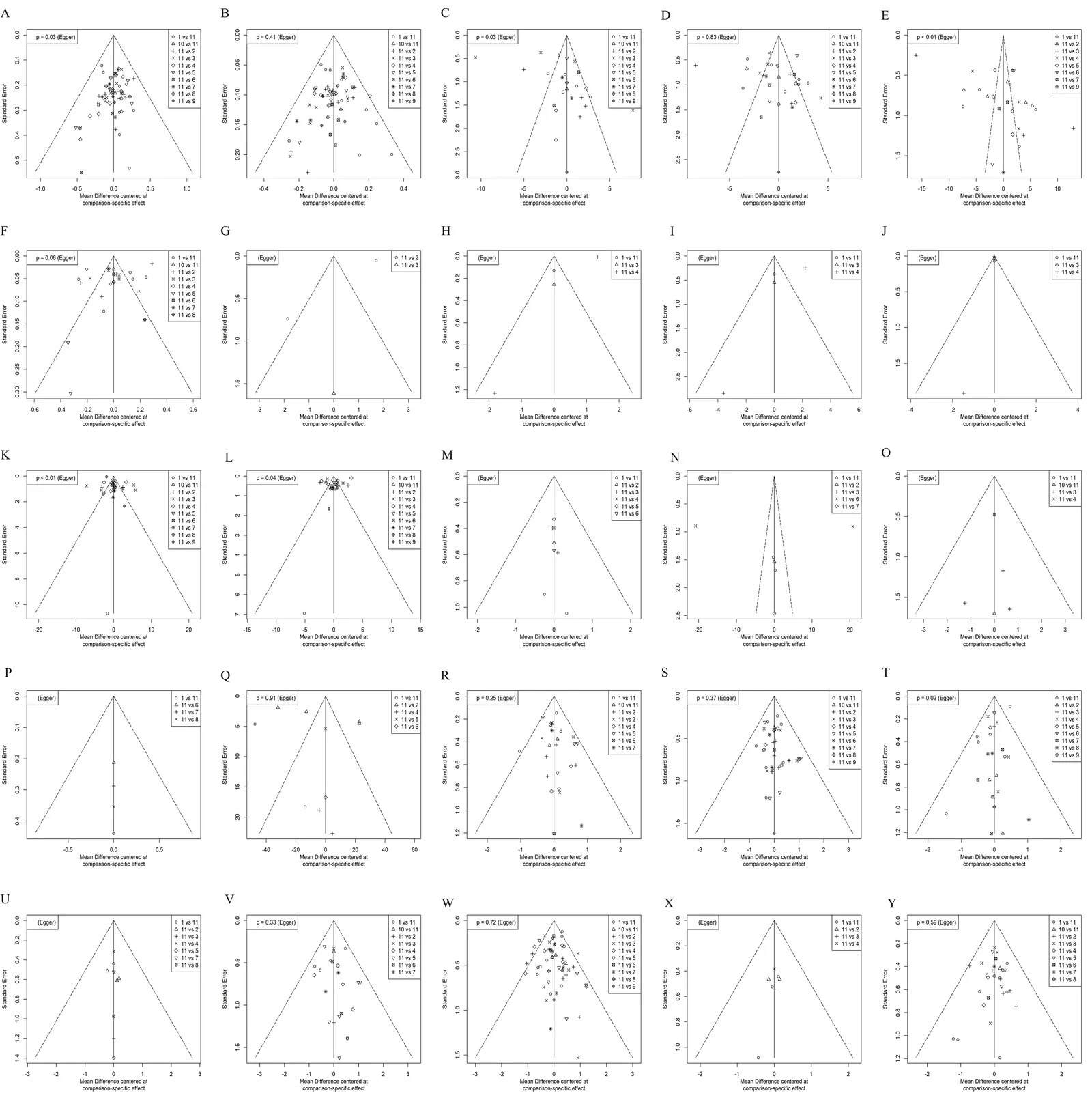
Calibrated Funnel Plots of Various Outcome Indicators for TCMIs+CG in the Treatment of NSCLC 1. KLTI+CG; 2. KAI+CG; 3. SQFZI+CG; 4. ADI+CG; 5. FFKSI+CG; 6. SMI+CG; 7. YDZYRI+CG; 8. XAPI+CG; 9. RSDTI+CG; 10. HQDTI+CG; 11. CG; **(A)** ORR; **(B)** DCR; **(C)** CD3^+^ T Cell; **(D)** CD4^+^ T Cell; **(E)** CD8^+^ T Cell; **(F)** CD4^+^ T Cell/CD8^+^ T Cell; **(G)** NK Cell; **(H)** IgA; **(I)** IgG; **(J)** IgM; **(K)** CEA; **(L)** CYFRA21-1; **(M)** NSE; **(N)** CA125; **(O)** CA19-9; **(P)** SCC; **(Q)** VEGF; **(R)** Adverse reaction: Leukopenia; **(S)** Adverse reaction: Abnormal liver function; **(T)** Adverse reaction: Myelosuppression; **(U)** Adverse reaction: Skin reaction; **(V)** Adverse reaction: Abnormal renal function; **(W)** Adverse reaction: Gastrointestinal reaction; **(X)** Adverse reaction: Decreased hemoglobin; **(Y)** Adverse reaction: Decreased platelet.

Overall, publication bias was assessed by funnel plots and Egger’s test in this meta-analysis. Significant asymmetry was detected in multiple outcome indicators, suggesting an overall risk of publication bias (**Figure 10**). Limited by publication bias, the number and quality of included studies, the conclusions have certain limitations and should be interpreted with caution. Further large-sample, high-quality, multicenter prospective studies are warranted to validate the results.

#### 3.5.10 Meta-regression analysis

**Figure 11:**
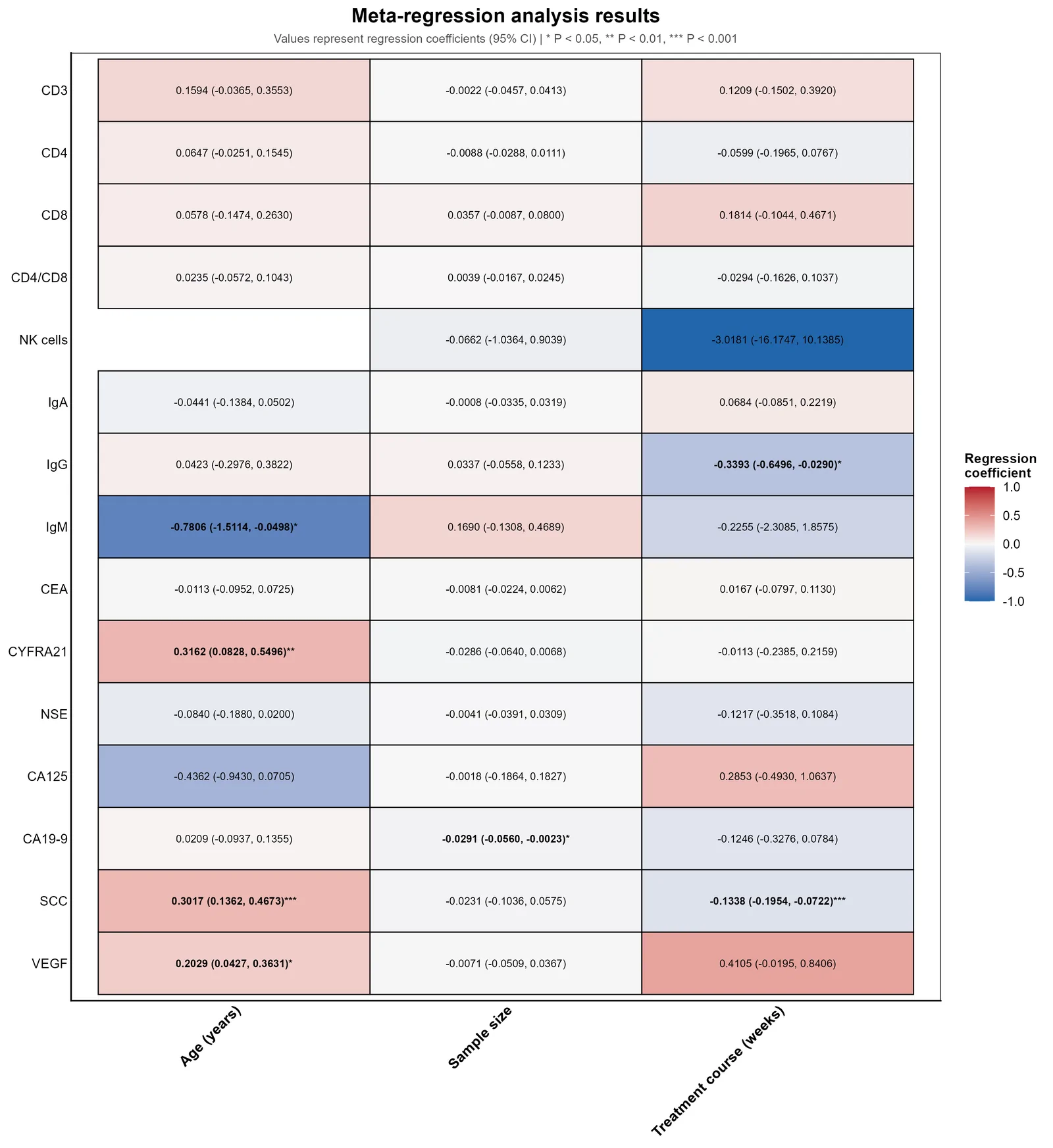
Meta-regression analyses of age, sample size, and treatment duration.

To explore potential sources of heterogeneity across studies, meta-regression analyses were performed using age, sample size, and treatment duration as covariates. The results showed that increasing age was positively associated with the effect sizes of CYFRA21-1, SCC, and VEGF (β=0.3162, 0.3017, and 0.2029, respectively; all P<0.05), whereas the effect size of IgM decreased with age (β=-0.7806, P<0.05). Sample size did not significantly influence most outcomes, except for a slight negative association with CA19–9 effect size (β=-0.0291, P<0.05). Longer treatment duration was associated with decreased effect sizes of IgG (β=-0.3393, P<0.05) and SCC (β=-0.1338, P<0.001), while no significant associations were observed for other indicators. Overall, age, sample size, and treatment duration may partially explain the heterogeneity of effect sizes for certain immune and tumor markers, whereas the remaining outcomes remained relatively robust (**Figure 11**).

However, substantial heterogeneity remained for several outcomes despite these exploratory analyses. For example, the heterogeneity of immune-related indicators and tumor markers remained considerable, with 12 values exceeding 90% for CD3+ T cells, CD4+ T cells, CD8+ T cells, CEA, andCYFRA21-1. Since meta-regression analyses were conducted using aggregate study-level data and included only a limited number of covariates, the identified associations should be interpreted cautiously and cannot establish causal explanations for the observed heterogeneity.

#### 3.5.11 Subgroup analysis

Among all outcome indicators, CYFRA21–1 was selected for subgroup analysis because a relatively large number of studies reported this outcome and substantial heterogeneity was observed in the pooled analysis. In contrast, the number of studies reporting other indicators was limited, making further subgroup analyses less reliable. Therefore, subgroup analyses were conducted for CYFRA21–1 according to sample size, treatment duration, chemotherapy regimen, and age to explore potential sources of heterogeneity.

**Figure 12:**
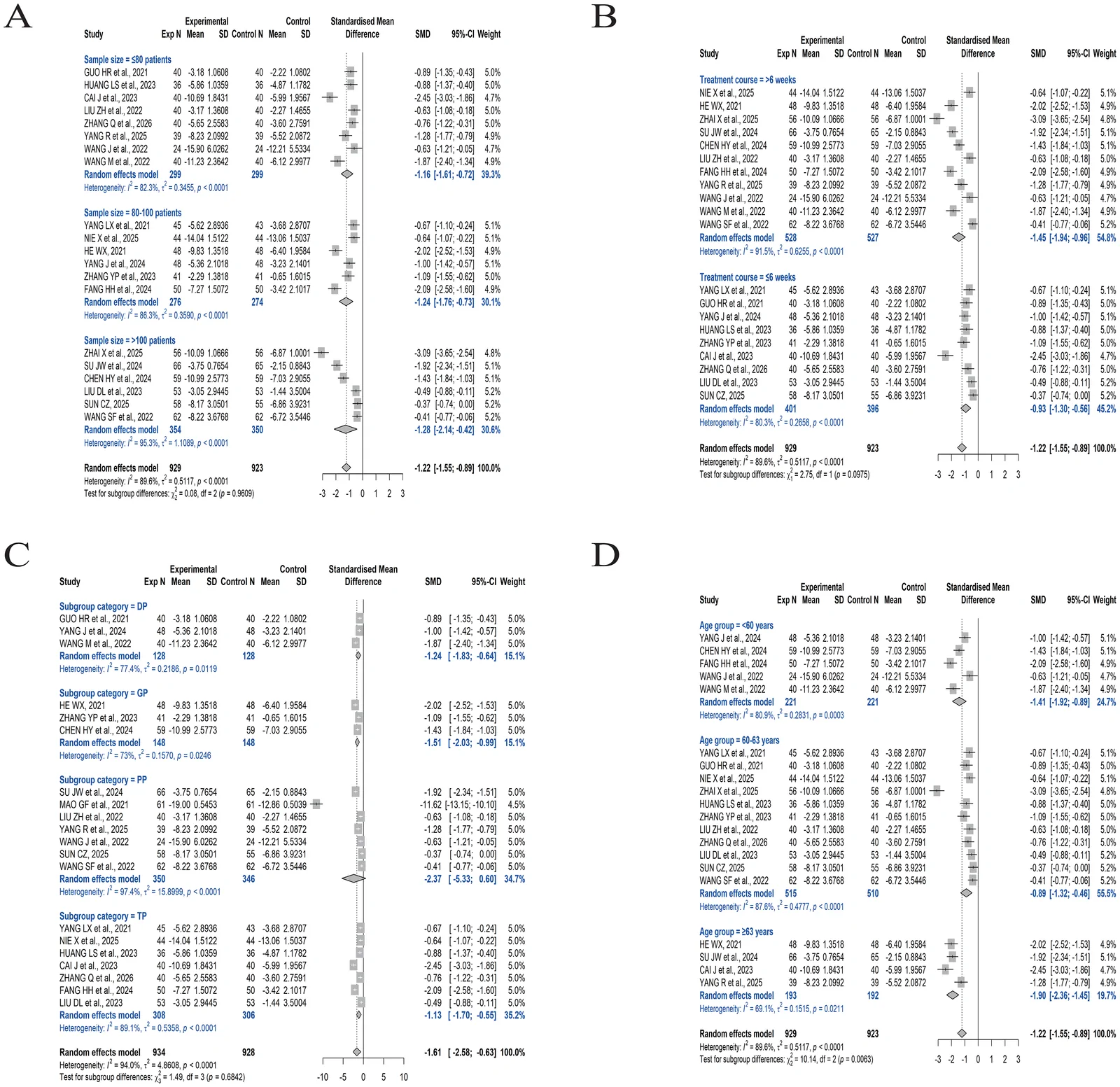
Forest plots of subgroup analyses for CYFRA21-1. **(A)** sample size, **(B)** treatment duration, **(C)** chemotherapy regimen, **(D)** age.

When stratified by sample size, the pooled effect sizes for ≤80, 80–100, and >100 patients were SMD=-1.16 (95% CI: -1.61 to -0.72), SMD=-1.24 (95% CI: -1.76 to -0.73), and SMD=-1.28 (95% CI: -2.14 to -0.42), respectively, all showing significant reductions in CYFRA21–1 levels compared with the control group (all P<0.05). No significant differences were observed among subgroups (χ²=0.08, P = 0.9609) (**Figure 12A**).

When stratified by treatment duration, the pooled effect sizes for ≤6 weeks and >6 weeks were SMD=-0.93 (95% CI: -1.30 to -0.56) and SMD=-1.45 (95% CI: -1.94 to -0.96), respectively. Both subgroups showed significant reductions in CYFRA21–1 levels (P<0.05), and no significant subgroup difference was detected (χ²=2.75, P = 0.0975) (**Figure 12B**).

When stratified by chemotherapy regimen, the pooled SMDs for the DP, GP, and TP regimens were -1.24 (95% CI: -1.83 to -0.64), -1.51 (95% CI: -2.03 to -0.99), and -1.13 (95% CI: -1.70 to -0.55), respectively, all demonstrating significant reductions in CYFRA21–1 levels (P<0.05). The pooled effect size for the PP regimen was SMD=-2.37 (95% CI: -5.33 to 0.60), which was not statistically significant. No significant differences were observed among chemotherapy regimens (χ²=1.49, P = 0.6842) (**Figure 12C**).

When stratified by age, the pooled effect sizes for patients aged <60 years, 60–63 years, and ≥63 years were SMD=-1.41 (95% CI: -1.92 to -0.89), SMD=-0.89 (95% CI: -1.32 to -0.46), and SMD=-1.90 (95% CI: -2.36 to -1.45), respectively, all showing significant reductions in CYFRA21–1 levels (P<0.05). Significant differences were identified among age subgroups (χ²=10.14, P = 0.0063), suggesting that age may be an important contributor to heterogeneity, with the greatest effect observed in patients aged ≥63 years (**Figure 12D**).

Overall, subgroup analyses based on sample size, treatment duration, and chemotherapy regimen revealed consistent treatment effects with no significant subgroup differences. In contrast, significant differences were observed among age subgroups, indicating that age may be a major source of heterogeneity in the CYFRA21–1 analysis.

Although subgroup analysis of CYFRA21-1suggested potential differences accordingto specific study characteristics, thesefindings should be interpreted as exploratorybecause the analysis was based on study-level aggregated data and was limited by thesmall number of available studies. Therefore,subgroup differences cannot establish causalrelationships.

#### 3.5.12 Confidence in network meta-analysis assessment of evidence certainty

In the present study, the CINeMA tool (82–84) was adopted to assess the certainty of evidence derived from the network meta-analysis. CINeMA comprehensively evaluates the reliability of evidence across six domains: within-study bias, across-study bias, indirectness, imprecision, heterogeneity, and incoherence. The results revealed that the certainty of evidence for most pairwise comparisons in this analysis was rated as low or very low.

Multiple contributing factors accounted for the downgraded evidence certainty. First, the overall methodological quality of included trials was unsatisfactory. Although randomization was explicitly mentioned in several studies, detailed descriptions of random sequence generation were absent in most trials; meanwhile, allocation concealment and blinding procedures were unreported in nearly all included publications, implying substantial risks of selection and performance bias. Second, discrepancies existed across trials regarding enrolled patients’ pathological subtypes, TNM staging regimens, chemotherapy protocols and treatment durations, which introduced clinical heterogeneity and compromised the comparability of pooled effect estimates across individual studies.

Furthermore, most evidence networks in this analysis exhibited a star-shaped topology with chemotherapy alone serving as the common comparator, whereas direct head-to-head comparisons between different traditional Chinese medicine injections (TCMIs) were lacking. Despite enabling indirect comparisons, such network structure precluded adequate verification of coherence between direct and indirect evidence, undermining the robustness of network-derived estimations. Accordingly, several SUCRA-based ranking outcomes should be interpreted as exploratory rather than definitive findings.

In addition, sparse included trials and limited sample sizes for certain outcome endpoints resulted in wide confidence intervals of pooled effect sizes, manifesting prominent imprecision. Moderate statistical heterogeneity was observed for several indicators, indicating between-study variability in therapeutic effects. Notably, all eligible studies were published in Chinese journals and conducted at single clinical centers within China, which may incur geographical and publication bias and further attenuate the certainty of relevant evidence.

In conclusion, notwithstanding the suggestive benefits of combining traditional Chinese medicine injections with conventional chemotherapy in enhancing treatment efficacy, improving immune function and alleviating adverse reactions, the overall certainty of available evidence remains limited. Well-designed, adequately powered, multicenter international randomized controlled trials with direct head-to-head comparisons are warranted in future investigations to upgrade evidence certainty, validate the genuine inter-agent differences among various TCMIs, and generate robust evidence to guide individualized clinical management.

## 4 Summary and discussion

### 4.1 Overall efficacy of TCMIs combined with chemotherapy for NSCLC

In recent years, increasing attention has been paid to the potential role of TCMIs as adjunctive therapies for advanced NSCLC. Although numerous randomized controlled trials have investigated the efficacy and safety of different TCMIs combined with chemotherapy, the comparative effectiveness among various TCMI-based regimens remains uncertain due to the lack of direct head-to-head comparisons. In this context, the present Bayesian network meta-analysis integrated available direct and indirect evidence to provide a comprehensive comparison of different TCMIs combined with chemotherapy and to explore their relative clinical benefits and safety profiles.

A total of 71 randomized controlled trials involving 6,386 patients with NSCLC were included in this study. The efficacy and safety of 10 TCMIs combined with chemotherapy were systematically compared. The results demonstrated that, compared with chemotherapy alone, TCMIs combined with chemotherapy generally improved the ORR and DCR, enhanced immune function, reduced tumor marker levels, and alleviated chemotherapy-related adverse reactions to a certain extent. Furthermore, the SUCRA rankings suggested that different TCMIs exhibited distinct advantages across various outcome measures, indicating that their therapeutic effects may not be limited to direct antitumor activity but may also involve immunomodulation, improvement of the tumor microenvironment, and attenuation of chemotherapy-induced toxicity.

NSCLC is a highly heterogeneous malignancy whose initiation and progression involve multiple biological processes, including uncontrolled tumor cell proliferation, evasion of apoptosis, chronic inflammation, immune suppression, and remodeling of the tumor microenvironment (85). Although platinum-based doublet chemotherapy remains one of the major treatment options for advanced NSCLC, its clinical benefits are often limited by chemotherapy resistance, impaired immune function, and treatment-related toxicities (86–88).

Therefore, improving immune status and treatment tolerance while maintaining effective tumor control has become an important goal in the comprehensive management of advanced NSCLC. The findings of this study suggest that TCMIs combined with chemotherapy may exert their therapeutic benefits through a sequential and integrated process involving suppression of tumor progression, restoration of antitumor immunity, reduction of tumor burden, and mitigation of treatment-related toxicity.

### 4.2 Tumor response and disease control: reflecting inhibition of tumor proliferation and progression

ORR and DCR are important indicators for evaluating short-term therapeutic efficacy in advanced NSCLC. ORR primarily reflects the degree of tumor shrinkage after treatment and represents the direct inhibitory effect on tumor cell proliferation and tumor burden. In contrast, DCR further reflects disease stabilization and delayed progression, providing a more comprehensive assessment of long-term control over tumor growth and invasion. In the present study, KLTI combined with chemotherapy showed a relatively high ranking probability for ORR, whereas RSDTI combined with chemotherapy demonstrated certain advantages in terms of DCR.

These findings suggest that different TCMIs may exert distinct therapeutic effects on tumor control in NSCLC. KLTI may primarily enhance the direct cytotoxic and inhibitory effects of chemotherapy on tumor cells, thereby achieving superior tumor response rates. In contrast, RSDTI may exert its benefits mainly by improving immune status, delaying disease progression, and maintaining disease stability, which may explain its advantage in DCR. For patients with NSCLC, clinical benefit is not solely reflected by marked tumor shrinkage. Even in the absence of complete or partial response, prolonged disease stabilization and delayed progression remain clinically meaningful outcomes.

From a mechanistic perspective, the development and progression of NSCLC are closely associated with aberrant activation of signaling pathways such as PI3K/Akt, MAPK, NF-κB, and JAK/STAT, which are involved in tumor cell proliferation, apoptosis resistance, inflammatory responses, and the development of chemotherapy resistance (89, 90). The TCMIs included in this study may regulate these signaling pathways through multiple targets from different perspectives. With regard to inhibiting tumor cell migration and invasion, ADI has been reported to inhibit the migration and invasion of gefitinib-resistant NSCLC cells by regulating the PLAT/FAK/AKT pathway (91). XAPI may downregulate the CCR5-CCL5 axis and downstream RhoC expression, inhibit FAK phosphorylation, and reduce extracellular matrix degradation mediated by MMP-2 and MMP-9. Furthermore, XAPI may downregulate HDGF expression and interfere with the IL-4/JAK1/STAT3 signaling crosstalk between NSCLC cells and tumor-associated macrophages, thereby inhibiting macrophage polarization toward the pro-tumor M2 phenotype (92). In terms of regulating apoptosis and enhancing chemotherapy sensitivity, FFKSI has been reported to regulate apoptosis by downregulating the PI3K/Akt signaling pathway (93). SMI combined with cisplatin may remodel mitochondrial dynamics in lung adenocarcinoma cells by upregulating Mfn2, downregulating ATAD3A to promote mitochondrial fusion, and upregulating Bax and activated caspase-3 while downregulating Bcl-2, thereby significantly enhancing cisplatin-induced apoptosis (94). Additionally, YDZYRI may regulate the JAK-STAT signaling pathway, alleviate oxidative stress injury, and enhance T-lymphocyte immune function (95). HQDTI may inhibit tumor cell growth by reducing Survivin protein expression and enhance the antitumor activity of PD-1^+^; CD8^+^; T cells by modulating mitochondrial metabolism (96, 97). Therefore, the observed improvements in ORR and DCR with TCMIs combined with chemotherapy may not simply result from increased chemotherapy intensity, but rather from synergistic interactions between direct cytotoxic effects, signaling pathway regulation, and immune microenvironment remodeling.

### 4.3 Improvement of immune function: a key component in the treatment of advanced NSCLC

The progression of NSCLC is not driven solely by uncontrolled tumor cell proliferation and metastasis; immune suppression also plays a non-negligible role in this process (98). During tumor evolution, cancer cells can impair immune surveillance through multiple mechanisms, including the secretion of immunosuppressive cytokines, recruitment of regulatory immune cells, upregulation of immune checkpoint molecules, and remodeling of the tumor microenvironment (99, 100).

Against this immunopathological background, the levels of CD3^+^; T cells, CD4^+^; T cells, CD8^+^; T cells, and NK cells serve as important indicators for assessing the status of tumor immune suppression. Specifically, CD3^+^; T cells reflect the overall T-lymphocyte population; CD4^+^; T cells play a central role in initiating and sustaining antitumor immune responses, and their recovery suggests restoration of helper T-cell functional reserves; CD8^+^; T cells, as the primary effector cells, directly mediate tumor cell elimination, and an increase in their numbers may indicate enhanced cytotoxic activity; NK cells constitute the first line of innate immune defense, capable of recognizing and killing malignant cells in the absence of antigen presentation. Furthermore, normalization of the CD4^+^;/CD8^+^; ratio carries greater clinical significance than changes in any single cell subset, as it reflects the reconstruction of the immune regulatory network. Changes in the levels of immunoglobulins IgA, IgG, and IgM reflect the status of humoral immune responses. Collectively, these indicators closely correspond to the immunopathological processes involved in NSCLC.

In clinical practice, patients with NSCLC commonly present with reduced levels of CD3^+^; T cells, CD4^+^; T cells, and NK cells, accompanied by an imbalanced CD4^+^;/CD8^+^; ratio, indicating impaired antitumor immune function (10, 101). In the present study, treatment with TCMIs led to varying degrees of improvement in the aforementioned indicators, suggesting that their value in advanced NSCLC extends beyond enhancing chemotherapy-induced tumor cell killing, and should rather be regarded as an important means of rebuilding host antitumor immunity. The favorable performance of SQFZI and RSDTI in immune-related outcomes indicates that these agents may reinforce long-term tumor control by promoting T-lymphocyte proliferation, enhancing NK cell activity, modulating immunoglobulin levels, and alleviating immunosuppression.

With regard to the underlying mechanisms, *in vivo* experiments demonstrated that SQFZI significantly reduced the proportions of MDSCs and Tregs in the tumor microenvironment, while concurrently increasing CD4^+^; and CD8^+^; T cell levels. The mechanistic basis may involve interference with fatty acid metabolism, specifically, through inhibition of arachidonic acid metabolism in melanoma cells, downregulation of D6D expression, and suppression of COX2 activity, leading to reduced production of the downstream product PGE_2_, thereby remodeling the tumor immune microenvironment and ultimately enhancing the antitumor efficacy of PD-L1 antibodies (102). Network pharmacology analyses further suggested that the potential mechanism by which RSDTI improves tumor immune function may be related to modulation of the PD-1/PD-L1 signaling pathway (103).

The aforementioned immunomodulatory effects may explain why certain TCMIs exhibit superior performance in terms of disease control rate and immune parameters, yet do not necessarily achieve the highest objective response rate. However, in interpreting these immune-related findings, the inherent limitations of peripheral blood immunophenotypic markers should be carefully considered. Lymphocyte subsets and immunoglobulin levels in peripheral blood merely reflect immune components within the circulatory system; they cannot fully represent the local immune status of the tumor microenvironment, nor can they distinguish genuine tumor-specific immune activation from nonspecific systemic immune recovery. Moreover, these markers are susceptible to confounding factors such as infections, concomitant medications, and interindividual baseline differences, and their dynamic changes do not necessarily correlate directly with the true functional status of tumor-infiltrating lymphocytes. Therefore, the observed improvements in immune parameters in this study should be prudently interpreted as preliminary signals of treatment-induced immune recovery, rather than as conclusive evidence of definitive antitumor immune activation. More definitive conclusions await validation through future studies incorporating tumor tissue biopsy, multiplex immunohistochemistry, and functional T-cell assays.

### 4.4 Reduction of tumor markers: reflecting changes in tumor burden, invasiveness, and the tumor microenvironment

Tumor markers serve as important auxiliary indicators for assessing disease activity and therapeutic response in NSCLC. The present study demonstrated that FFKSI ranked highly in reducing CEA and VEGF, whereas SQFZI showed favorable performance in improving NSE and CA19–9 levels. In addition, RSDTI exhibited certain advantages in reducing CYFRA21-1.

Different tumor markers reflect distinct biological processes involved in the development and progression of NSCLC. CEA is commonly associated with tumor burden, metastatic potential, and disease progression in lung adenocarcinoma. CYFRA21-1, derived primarily from cytokeratin fragments, is often elevated in association with tumor cell destruction, squamous cell carcinoma–related pathological activity, and disease progression. NSE is associated with neuroendocrine differentiation and tumor aggressiveness. Elevated levels of CA125 and CA19–9 may indicate increased tumor burden, pleural involvement, or advanced disease activity, whereas SCC is commonly used to reflect pathological changes related to lung squamous cell carcinoma (104, 105). The reductions observed in these tumor markers suggest that combination therapy may decrease tumor burden and attenuate malignant biological behavior. Among these markers, VEGF deserves particular attention. Sustained tumor growth and distant metastasis are highly dependent on angiogenesis, and VEGF is one of the key mediators driving tumor neovascularization. Elevated VEGF levels promote the formation of new blood vessels, improve tumor blood supply, and create favorable conditions for tumor invasion and metastasis (106). However, recent studies have further revealed complex immune crosstalk between VEGF signaling and immune checkpoint inhibition. In cholangiocarcinoma models, VEGF blockade combined with CTLA-4 and PD-L1 antibodies induces a B-cell activating factor (BAFF)-dependent multimodal immune effect, promoting pro-inflammatory B-cell responses and subsequently driving Treg expansion and remodeling toward an antitumor Th1-like fragile phenotype via BAFF- and IL-12-mediated pathways (107). This finding suggests that VEGF signaling may play a critical regulatory role in maintaining the immunosuppressive function of Tregs. Therefore, a reduction in VEGF levels may have dual significance: on the one hand, it reduces tumor angiogenesis; on the other, it alleviates VEGF-mediated immunosuppression and may restore host antitumor immune surveillance by remodeling the functional phenotype of Tregs. Accordingly, the superior performance of FFKSI in reducing VEGF suggests that its therapeutic effect may extend beyond inhibiting tumor angiogenesis and lowering tumor marker levels. It may also serve as an important mechanism for reestablishing host antitumor immunity by relieving VEGF-mediated immunosuppression and remodeling the immune microenvironment.

### 4.5 Reduction of chemotherapy-related toxicity: improving treatment tolerability and continuity

Safety remains a critical consideration in the management of advanced NSCLC. The present study demonstrated that the addition of TCMIs to chemotherapy did not increase the overall risk of adverse events. On the contrary, several TCMI-based regimens appeared to reduce chemotherapy-related toxicities, including leukopenia, myelosuppression, gastrointestinal reactions, hepatic and renal dysfunction, hemoglobin reduction, and thrombocytopenia.

Although chemotherapeutic agents effectively eliminate rapidly proliferating tumor cells, they inevitably damage normal tissues with high proliferative activity, such as hematopoietic cells in the bone marrow, gastrointestinal mucosal cells, and hepatic and renal tissues. Leukopenia and myelosuppression may increase the risk of infection and further compromise immune function. Decreases in hemoglobin and platelet counts can result in fatigue, increased bleeding risk, and reduced treatment tolerance. Gastrointestinal toxicities may impair nutritional intake and patient adherence, whereas hepatic and renal dysfunction may limit subsequent treatment options. Consequently, treatment-related adverse events not only negatively affect patients’ quality of life but may also lead to chemotherapy dose reductions, treatment delays, or even premature discontinuation.

Several TCMIs included in this study demonstrated favorable effects in reducing chemotherapy-induced toxicities, suggesting that they may improve treatment tolerance through multiple mechanisms, including protection of hematopoietic function, attenuation of inflammatory injury, alleviation of gastrointestinal symptoms, and enhancement of tissue repair capacity. For patients with NSCLC, the concept of “toxicity reduction” extends beyond a simple safety benefit and may ultimately translate into improved treatment adherence, greater completion of planned chemotherapy cycles, and more stable overall therapeutic outcomes.

Therefore, the clinical value of combining TCMIs with chemotherapy may lie not only in improving short-term antitumor efficacy but also in supporting patients’ ability to sustain long-term treatment. This aspect is particularly important in NSCLC, where maintaining treatment continuity is often essential for maximizing clinical benefit.

### 4.6 Differential advantages of various traditional Chinese medicine injections and their clinical implications

The findings of this study indicate that different TCMIs exhibit distinct advantages across various outcome measures, and no single intervention demonstrated absolute superiority in all evaluated endpoints. For example, KLTI appeared to be more effective in improving ORR, whereas RSDTI showed advantages in DCR and several T-cell–related immune indicators. SQFZI demonstrated favorable performance in NK-cell activity, IgG levels, and certain tumor markers, while FFKSI showed notable advantages in reducing CEA, VEGF, and several chemotherapy-related adverse events.

These findings suggest that SUCRA rankings should be interpreted cautiously and should not be used as the sole basis for comparing or selecting TCMIs. Instead, the observed differences among TCMIs should be regarded as exploratory information that requires integration with additional clinical evidence and further validation. For patients with a high tumor burden who require rapid tumor shrinkage and improved short-term response, greater attention may be given to regimens with superior ORR performance. In contrast, for patients with impaired immune function, poor treatment tolerance, or a clinical need for prolonged disease stabilization, TCMIs with stronger immunomodulatory and toxicity-reducing effects may be more appropriate. Similarly, for patients presenting with markedly elevated tumor markers such as VEGF or CEA, which may indicate an active tumor microenvironment or enhanced angiogenesis, TCMIs demonstrating favorable effects on these biomarkers may deserve particular consideration.

Therefore, the current findings should be considered as exploratory evidence regarding the potential differential characteristics of TCMIs rather than direct guidance for personalized treatment selection. These results may provide a reference for future studies investigating whether specific patient characteristics are associated with differential responses to individual TCMIs.

Future studies should further explore the populations most likely to benefit from each TCMI and identify their optimal clinical application scenarios, thereby facilitating more precise and evidence-based treatment selection.

To further investigate the potential sources of heterogeneity, meta-regression analyses were performed across all eligible immune and tumor-marker outcomes. The results suggested that age may be associated with variations in the effect sizes of CYFRA21-1, SCC, VEGF, and IgM, whereas sample size and treatment duration appeared to influence only a limited number of outcomes. These findings indicate that patient-related and study-level characteristics may partially contribute to the observed between-study heterogeneity. Because CYFRA21–1 was among the most frequently reported tumor markers and demonstrated substantial heterogeneity in the pooled analysis, additional subgroup analyses were conducted for this outcome. The subgroup findings were generally consistent with the meta-regression results, showing significant differences across age categories but not across sample size, treatment duration, or chemotherapy regimen subgroups. This suggests that age may be statistically associated with heterogeneity in CYFRA21–1 outcomes within the included studies. Nevertheless, both meta-regression and subgroup analyses were based on aggregate study-level data rather than individual patient data; therefore, these findings should be considered exploratory and interpreted cautiously.

### 4.7 Limitations

Several limitations of the present study should be acknowledged when interpreting the findings.

First, the overall methodological quality of the included studies was poor, which represents a major limitation of the present analysis. Although some trials reported the use of randomization, many either used inadequately described methods or did not provide sufficient details regarding random sequence generation. None of the 71 included RCTs reported allocation concealment or blinding procedures, and no prospectively registered trial protocols were available. These methodological deficiencies raise substantial concerns regarding selection bias, performance bias, detection bias, and selective reporting across the entire evidence base. In addition, most studies had relatively small sample sizes, which may have resulted in unstable effect estimates and further reduced confidence in the findings. According to the CINeMA assessment, the certainty of evidence for most comparisons was rated as very low, mainly because of within-study bias, potential reporting bias, substantial heterogeneity, imprecision, and the predominance of indirect evidence. Therefore, the observed effect estimates and SUCRA rankings should not be regarded as confirmatory evidence of the comparative superiority of specific TCMIs. Rather, the findings should be interpreted as exploratory and hypothesis-generating and should not be used as a direct basis for clinical decision-making. Confirmation in prospectively registered, adequately powered, multicenter RCTs with rigorous randomization, allocation concealment, appropriate blinding, and standardized outcome reporting is required.

Second, considerable statistical and clinicalheterogeneity existed among the included studies. Several critical outcomes showed extremely high heterogeneity, with I^2^ values exceeding 90%, including CD3+ T cells, CD4+ T cells, CD8+ T cells, CEA, andCYFRA21-1. Such substantial heterogeneity reduces confidence in the pooled effect estimates and limits the generalizability of the findings. Although meta-regression and subgroup analyses were performed toexplore potential sources of heterogeneity, these analyses were based on aggregate study-level data and included only a limited number of covariates. Therefore, the observed associations should be considered exploratory rather than causal explanations. Moreover, residual confounding from unmeasured clinical and methodological factors may have contributed substantially to the observed heterogeneity. These factors may include baseline disease stage, pathological subtype, chemotherapy regimens, chemotherapy dosage, TCMI type and dosage, treatment cycles, concomitant therapies, and differences in outcome measurement methods. Since these variableswere inconsistently reported or unavailable in the included studies, their potential influencecould not be fully assessed.

Third, the immune markers assessed in this study were limited to routine peripheral blood counts, including the percentages and absolute counts of T lymphocyte subsets (CD3^+^;, CD4^+^;, CD8^+^;) and NK cells, as well as the CD4^+^;/CD8^+^; ratio. Key immunoregulatory molecules, such as Tregs, MDSCs, immune checkpoint molecules (e.g., PD-1/PD-L1, CTLA-4), and cytokines (e.g., IL-2, IL-10, IFN-γ, TGF-β), were not evaluated in the included studies. The absence of these indicators represents an important gap, as they are critical to understanding the potential immunomodulatory mechanisms of TCMIs. The original attribution of this gap solely to “a limited number of studies” is insufficient. In fact, even among the available studies, these markers were not routinely measured or reported, which may reflect both methodological constraints and the exploratory nature of the current evidence base. Therefore, the present findings provide only a partial view of the immunomodulatory effects of TCMIs, and their ability to capture the full spectrum of immune regulation, particularly immune checkpoint-mediated pathways and Treg/MDSC-driven immunosuppression, is inherently limited.

Fourth, the evidence network included in this study was primarily characterized by a star-shaped structure, with limited direct head-to-head comparisons among different TCMIs. Consequently, the results of the network meta-analysis relied largely on indirect comparisons through shared control groups. Although Bayesian network meta-analysis improves comparative efficiency, the absence of sufficient closed loops prevented a comprehensive assessment of consistency between direct and indirect evidence. Therefore, the SUCRA rankings should be interpreted as reflecting relative probabilities of superiority rather than definitive differences in efficacy.

Fifth, treatment strategies for NSCLC have evolved rapidly in recent years, with targeted therapies and immune checkpoint inhibitors becoming important components of modern management. However, most studies included in the present analysis were conducted in the context of conventional chemotherapy and did not adequately evaluate the efficacy and safety of TCMIs when combined with immunotherapy or targeted therapy. Accordingly, the present findings are primarily applicable to patients with advanced NSCLC receiving chemotherapy-based treatment, and the role of TCMIs within contemporary multimodal treatment strategies requires further investigation.

Sixth, although multiple outcome measures were evaluated, including immune indicators and tumor markers, these variables are largely surrogate endpoints and cannot fully substitute for long-term clinical outcomes. Owing to the limited availability of data on overall survival (OS), progression-free survival (PFS), and quality of life (QoL), it remains uncertain whether the observed short-term advantages associated with TCMIs can ultimately be translated into meaningful long-term survival benefits.

Seventh, all included studies were conducted in China, and the vast majority were published in Chinese-language journals. This may reflect regional differences in clinical practice patterns and may also increase the likelihood of publication bias, given that studies reporting positive findings are generally more likely to be published. Furthermore, the number of studies available for several outcomes was relatively limited, reducing the precision of the evidence. This limitation was also reflected in the CINeMA assessment, in which the certainty of evidence for most comparisons was rated as very low.

Eighth, although meta-regression and subgroup analyses were conducted to explore potential sources of heterogeneity, the present study was still unable to evaluate treatment effects across several clinically important patient-level subgroups. For example, whether patients with different pathological subtypes (lung adenocarcinoma versus squamous cell carcinoma), driver mutation status, PD-L1 expression levels, or performance status derive differential benefits from TCMIs remains unclear because of insufficient evidence. Future studies should incorporate the principles of precision medicine to identify the populations most likely to benefit from individual TCMIs and to define their optimal clinical application settings, thereby enabling more personalized treatment decision-making. Moreover, although meta-regression and subgroup analyses were performed to explore potential sources of heterogeneity, these analyses were based on study-level aggregated data and included only a limited number of covariates. Therefore, the identified associations should be interpreted as exploratory findings rather than definitive explanations for heterogeneity. Residual confounding from unmeasured or inconsistently reported factors, including baseline disease characteristics, pathological subtype, chemotherapy regimens, TCMI types and dosages, treatment duration, and concomitant therapies, may have contributed substantially to the observed variability. Future studies using individual-patient-data meta-analysis or well-designed prospective trials are warranted to further clarify these potential modifiers.

Finally, although the present analysis did not identify an increased risk of adverse events associated with TCMIs combined with chemotherapy, the certainty of safety conclusions remains limited. The included studies generally lacked standardized adverse-event reporting, including consistent definitions, severity grading, duration, and management information. Therefore, the potential safety advantages observed in this study should be considered preliminary and require confirmation through future high-quality trials with standardized safety assessment.

## 5 Conclusion

In summary, TCMIs combined with chemotherapy maybe associated with improvements in clinical response, immune-related indicators, tumor-associated markers, and treatment tolerability in patients with advanced NSCLC. Different TCMIs may have distinct advantages across different outcomes, suggesting that their clinical application should be individualized according to patient characteristics and treatment goals. Meta-regression analysis suggested that age, sample size, and treatment duration may be potential sources of heterogeneity for some outcomes, with age potentially associated with heterogeneity in CYFRA21–1 results; this finding was further supported by subgroup analysis. However, owing to limitations in study quality, sample size, publication bias, heterogeneity, and the lack of long-term follow-up data, the findings should be interpreted with caution. Further well-designed, multicenter, large-scale randomized controlled trials are needed to validate the efficacy, safety, and optimal target populations of different TCMIs combined with chemotherapy.
